# Complex dynamics at the nanoscale in simple biomembranes

**DOI:** 10.1038/s41598-017-11068-5

**Published:** 2017-09-11

**Authors:** Nirod Kumar Sarangi, K. G. Ayappa, Jaydeep Kumar Basu

**Affiliations:** 1 Department of Physics, Indian Institute of Science, Bangalore, 560 012 India; 2 Department of Chemical Engineering, Indian Institute of Science, Bangalore, 560 012 India; 3 Center for Biosystems Science and Engineering, Indian Institute of Science, Bangalore, 560 012 India

## Abstract

Nature is known to engineer complex compositional and dynamical platforms in biological membranes. Understanding this complex landscape requires techniques to simultaneously detect membrane re-organization and dynamics at the nanoscale. Using super-resolution stimulated emission depletion (STED) microscopy coupled with fluorescence correlation spectroscopy (FCS), we reveal direct experimental evidence of dynamic heterogeneity at the nanoscale in binary phospholipid-cholesterol bilayers. Domain formation on the length scale of ~200–600 nm due to local cholesterol compositional heterogeneity is found to be more prominent at high cholesterol content giving rise to distinct intra-domain lipid dynamics. STED-FCS reveals unique dynamical crossover phenomena at length scales of ~100–150 nm within each of these macroscopic regions. The extent of dynamic heterogeneity due to intra-domain hindered lipid diffusion as reflected from the crossover length scale, is driven by cholesterol packing and organization, uniquely influenced by phospholipid type. These results on simple binary model bilayer systems provide novel insights into pathways leading to the emergence of complex nanodomain substructures with implications for a wide variety of membrane mediated cellular events.

## Introduction

In the cell membrane, cholesterol plays a key role in regulating various biophysical and biochemical processes. Specific interactions with other membrane lipids and proteins not only helps maintain both membrane structural integrity and fluidity but also modulates events such as signalling, transport and binding^1^. The distribution of cholesterol varies widely in mammalian cell membranes, with concentrations ranging from ~20–30%^2^ in the plasma membrane and ~1% in the endoplasmic reticulum (ER), of the total lipid content in the cell^3^. Cholesterol is widely believed to be the key driving force behind formation of compositionally and dynamically heterogeneous nanodomains in cell membranes, critical for various biological activities including signalling, viral infections and membrane trafficking^4–8^. In view of the widely accepted paradigm that biological membranes organize into domains of different compositions and sizes, both at the nano- and microscale, a large number of studies have focussed on investigating domain formation and phase separation in model phospholipid-cholesterol membranes using both experimental^9–11^ theoretical^12, 13^ and molecular dynamics^14, 15^ techniques. The current view emerging out of these studies is that domains are dynamic, heterogeneous structures rich in cholesterol and sphingomyelin (SM), ranging from ~10–200 nm in diameter, and present in the lipid membranes of all eukaryotic cells^8^.

In order to connect lipid structure and domain formation, model ternary systems involve a combination of high melting saturated lipids, a low melting unsaturated lipid and cholesterol^16^. In such systems, domain formation is related to the presence of co-existing phases in the phase separated regimes. However domain formation at micron and sub-micron scales have also been observed in simple binary phospholipid-cholesterol systems, and can arise from the coexistence of a liquid-ordered (*L*_*o*_) and a liquid-disordered (*L*_*d*_) phase within the membranes or even exist in the absence of distinct phase separation above the melting temperature of the given phospholipid^17^. Several independent techniques have been used to verify the presence of nanoscale domains in a variety of model bilayer systems, involving multiple lipid components and/or peptides and proteins^18–28^. For example, recently 50 nm lipid nanodomains with a domain lifetime of 220 ± 60 ms has been observed using interferometric scattering microscopy (iSCAT) microscopy in phase-separated DOPC:bSM(1:1) droplet interface bilayers^29^.

The intriguing possibility of observing nanoscale domain formation in the absence of distinct thermodynamic phase separation in minimal two-component phospholipid-cholesterol membranes, particularly at high cholesterol concentrations has recently received renewed attention^15, 30, 31^. Recent molecular simulation studies suggest that nanoscale domain formation can also exist within an otherwise homogeneous *L*_*o*_ and/or *L*_*d*_ phase indicating the emergence of complex nanoscale morphology driven by phospholipid-cholesterol interactions^14, 15, 32^. The key question which remains unanswered despite, at least, two decades of intense research is whether there exists a universal underlying physical principle to explain the emergence of nanoscale compositional and dynamical heterogeneity. In an attempt to explain the emergence of this phenomenon several models have been proposed^33–35^.

Obtaining microscopic insight on the causal connection between lipid composition and nanodomain formation has proven difficult, largely, due to the small length scales involved (10–100 nm). To probe the existence, origin and extent of such spatio-temporal biomembrane platforms at the nanoscale, super-resolution microscopy techniques, especially stimulated emission depletion based technique (STED)^36^, in combination with fluorescence correlation spectroscopy (STED-FCS)^37–41^ could provide a powerful means to correlate dynamics with local nanoscale membrane structures. STED-FCS studies on plasma membrane revealed complex nanoscale lipid dynamics attributed to the affinity of sphingomyelin to cholesterol^40^. This and more recent studies suggest the ability of STED-FCS to probe nanoscale dynamical heterogeneity of lipids in model membranes and potentially probe domain specific dynamics within length scales of 10’s of nm’s^41^.

Here, we report extensive studies on supported phospholipid bilayer platforms with variable phospholipid and cholesterol composition using STED-FCS to directly detect the presence and emergence of nanodomains in the simplest of two-component lipid bilayers. For this purpose we have chosen lipid bilayer systems consisting of either a low melting unsaturated 1,2-dioleoyl-*sn*-glycero-3-phosphocholine (DOPC) lipid, a low melting mono-unsaturated 1-palmitoyl-2-oleoyl-*sn*-glycero-3-phosphocholine (POPC) or a high melting saturated lipid 1,2-dimyristoyl-*sn*-glycero-3-phosphocholine (DMPC), in combination with cholesterol (Chl) with variable composition. At 50% cholesterol composition, we observe, a bimodal distribution of liquid-like diffusivities in confocal FCS, confirming the presence of heterogeneous lipid partitioning in an otherwise homogeneous bilayer. Strikingly, STED-FCS measurements reveals the existence of crossover at length scales of ~100–150 nm, in diffusion behavior within the spatially distinct regions as observed in confocal FCS. This dynamically distinct signature at the nanoscale has not been measured before. Further, the presence of Brownian/non-Brownian dynamics with or without a length-scale dependent crossover is dependent on the location of the domains in the dynamically distinct regions. At a lower cholesterol concentration of 33% only a weak evidence of non-Brownian dynamics is observed in DOPC and DMPC bilayers. Our results provide clear evidence of the rich level of nanoscale dynamical heterogeneity possible in two-component low melting lipid bilayers mediated by cholesterol and lipid saturation, with a direct and quantitative estimate of the length scales at which the heterogeneity exists. Since the experimentally observed dynamical crossover occurs without an underlying structural transition on the same length scale it is possible that this lipid dynamics is akin to glass-like dynamics recently reported using molecular dynamics simulations of a single component high melting gel-like phospholipid bilayer^33^. Further, our STED-FCS results on model membranes sheds light not only on the pathways for inducing nanosized proteolipid domains in eukaryotic cell membranes, but also opens up the feasibility of probing intra-domain lipid dynamics to obtain insight into various bio-membrane mediated processes occurring at the nanoscale.

## Results

### Cholesterol concentration dependent lipid dynamical heterogeneity

Supported lipid bilayers (SLBs) containing different lipids such as DOPC, POPC and DMPC with variable concentration of cholesterol were prepared using the Langmuir-Blodgett (LB) technique. The isotherms of cholesterol/phospholipid mixed monolayer did not show demixing, and the successful transfer of two consecutive monolayers were performed at a holding surface pressure of 35 mN/m (Fig. S1, SI) with a transfer ratio of 1 ± 0.1. Optical imaging and fluorescence correlation spectroscopy (FCS) measurements were carried out within 4–5 h of the LB transfer at 24 ± 2 °C. At 25 and 33% cholesterol, confocal and STED microscopy of the SLBs revealed a fairly homogeneous bilayer with uniform distribution of fluorescent intensity of Atto488-PE stained prior to bilayer fabrication (Figs S2–4, SI). Lipid diffusion is a commonly used parameter to detect distinct co-existing phases. Fluorescence correlation spectroscopy (FCS) revealed a unimodal lipid diffusion (for fitting procedure and analysis of FCS, see Fig. S5, SI) indicative of the absence of any phase separation for the SLBs at these intermediate compositions of cholesterol (Fig. S6, SI). The mean lipid diffusivity, *D* (refer Eq. S3 in SI), decreased in bilayers going from DOPC (highest) to DMPC (lowest) for a given cholesterol concentration and generally decreased linearly with increasing cholesterol concentration for a given lipid consistent with earlier measurements^42^. The generally accepted phase diagram for binary systems at intermediate cholesterol concentrations indicate a co-existence of *L*_*d*_ and *L*_*o*_ phases^43, 44^. Although we do not find any evidence of coexisting phases in our confocal and FCS diffusivity measurements at these concentrations, our results do not necessarily invalidate the proposed thermodynamic phase diagram. It might only indicate that the spatial extent of the co-existing phases if present lies below the detection limits of the optical techniques used in our measurements. The absence of distinct phase separation into *L*_*o*_ and *L*_*d*_ phases in our images has been recently proposed as an alternative to the traditional co-existence view^17, 45^. This picture is largely consistent with reports for DOPC and POPC membranes with cholesterol where phase co-existence was not detected using pulse field gradient, NMR diffusivity measurements^46, 47^ with only a weak diffusivity contrast observed around 300 K for DMPC. Most of the studies which determine phase diagrams are based on measurements which do not have spatial resolution and domains if present are estimated to lie below the optical detection limit^46^. Further, optical microscopy measurements which reveal the co-existing phases at this composition and measurement temperature used in our study are absent.

**Figure 1:**
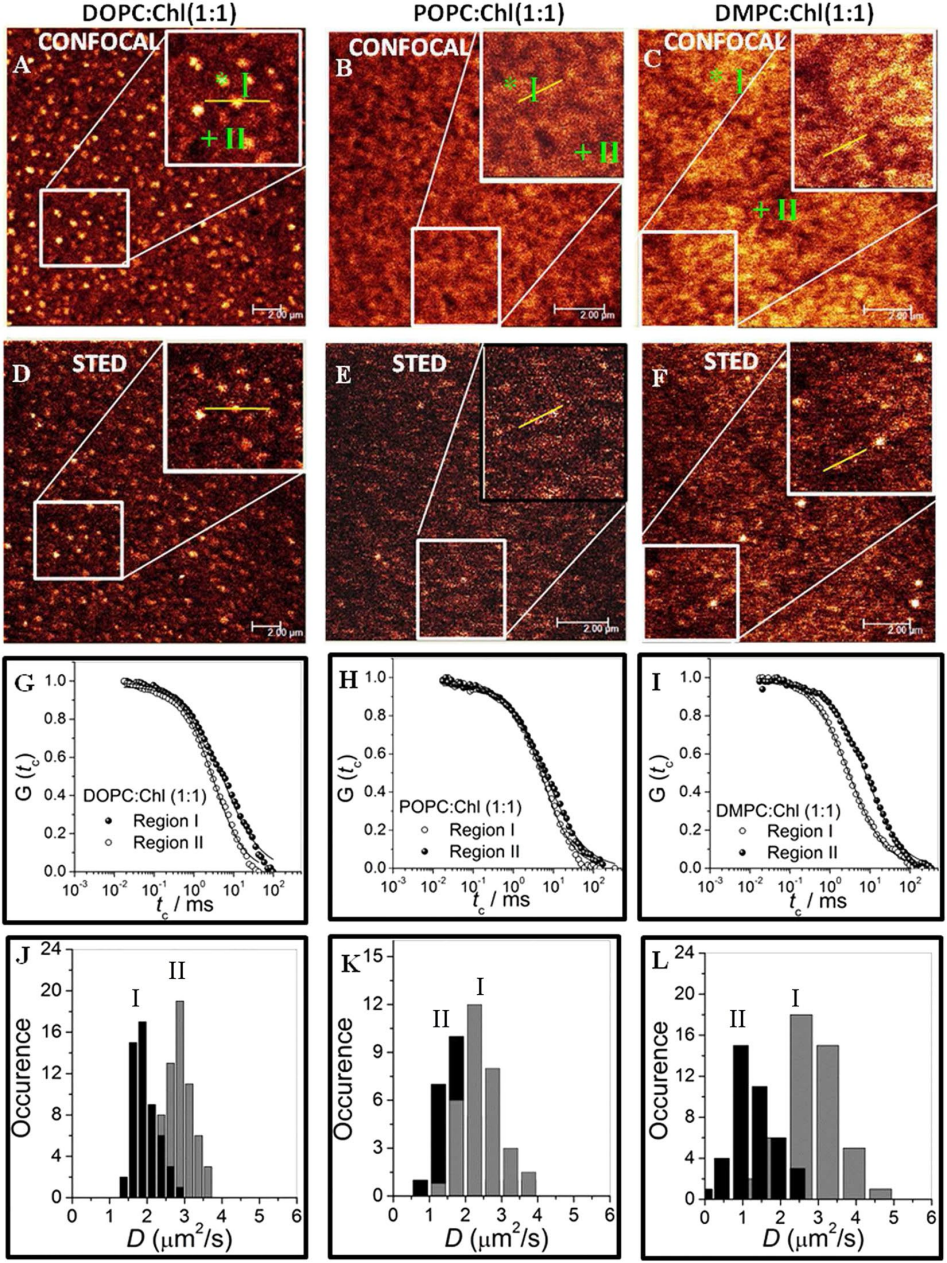
Figures (**A**–**C**) shows the confocal microscopy image of DOPC, POPC and DMPC containing 50% cholesterol in the bilayer. The brighter regions (region I) are dye rich marked in ‘*’ and the darker region (region II) are dye poor, marked with ‘+’. Figure (**D**–**F**) are the respective STED microscopy images collected at same region at a maximum power of 260 mW with expected PSF of 80 nm. Inset shows the zoomed image of selected regions as shown in the rectangle box. All images were 512 × 512 pixels, line average was set at 2 and scan speed at 600 Hz. In all the images, the bilayers are stained with Atto488-PE (0.0005 mol%). The scale bar is 2 *μ*m. Representative FCS correlation curves are shown in (**G**) DOPC:Chl (1:1), (**H**) POPC:Chl (1:1) and (**I**) DMPC:Chl (1:1) bilayers. Panel J, K and L are the respective histograms of diffusion coefficient (**D**) measured from the respective spatial points marked as ‘I’ and ‘II’ in (**A**–**C**). The data in (**J**–**L**) are collected from ≈30–50 measurements each corresponding to type I and II regions from three independent bilayers. All measurements were performed within 4–5 h of the LB transfer at 24 ± 2 °C.

On the other hand for SLBs with 50% cholesterol the situation changes dramatically and both confocal and STED images (Fig. 1), reveal the appearance of domain formation on the scale of 200–600 nm in these bilayers, distinctly visible in both DOPC and DMPC systems (see line profile analyses, Fig. S7a–c, SI), while it is much weaker in POPC. Various studies suggest that most lipid bilayers at such high cholesterol content should be in a homogeneous *L*_*o*_ phase^45, 48^. However, there are also reports which suggest the presence of cholesterol induced microstructure in the *L*_*o*_ phase at high cholesterol content^31, 49^ before the onset of crystallization. Nevertheless, a bimodal distribution of lipid diffusivities, emerge in these SLBs as revealed by confocal FCS (Fig. 1G–L). The difference in diffusivities between the two emerging sub-populations is larger for the symmetric (two long fatty acid chains with equal number of carbon atoms, *C*_*n*_) lipids DOPC (*C*_18_) and DMPC (*C*_14_) when compared with the asymmetric POPC (unequal number of carbon atoms in fatty acid chain, *C*_18_ and *C*_16_) where the sub-populations are not clearly separated.

Since domain formation is driven by the addition of cholesterol, it is reasonable to expect an inhomogeneous distribution of cholesterol in the two emerging populations at 50% cholesterol. Based on the values of the lipid diffusivities, *D* we identify and henceforth refer to the high *D* regions as *F* while the low *D* regions will be called *S*. For DOPC, *F* corresponds to cholesterol poor regions (regions II in Fig. 1) while for DMPC, *F* corresponds to cholesterol rich regions (regions I in Fig. 1). This distinction is not that easily identifiable for POPC but tentatively regions I corresponds to *F* for POPC. In addition we would also like to point out that the Atto488-PE as the fluorescent dye marker for our bilayer membranes does not partition exclusively to cholesterol rich or cholesterol poor phases. Complementary cholesterol extraction experiments with *β*-cyclodextrin (CD) (see Figs S8–10, SI) clearly shows a differential extraction rate for cholesterol from the regions *S* and *F*, supporting our conclusions that these phases correspond to cholesterol-rich and cholesterol-poor regions respectively for DOPC and vice versa for DMPC. Additionally, at a low cholesterol content (25%) depletion of cholesterol is faster for DOPC and moderate for DMPC bilayers (cf. Figs S9 a and S11, SI) suggesting that cholesterol reorganization and binding is a function of phospholipid-cholesterol interactions. To summarize our observations from microscopy images and FCS data in confocal mode with a spatial resolution of ~200 nm, we observe large scale (200–600 nm, cf. Fig. S7d–f, SI) heterogeneities in membrane lipid dynamics for the highest cholesterol concentration of 50%. At all concentration below this, the membrane appear largely homogeneous in imaging and unimodal diffusivity in confocal based measurement. Further, we would like to point out that the highest concentration of cholesterol studied in our work is well below the solubility limit of cholesterol for two-component binary phospholipid-cholesterol mixtures. It has also been suggested that a solubility limit of cholesterol in DOPC bilayers is 0.67^13, 50, 51^, above which cholesterol precipitates as monohydrate crystals^52^, or forms immiscible cholesterol bilayer domains^53^ due to demixing. The possibility of demixing can be discarded due to the fact that there is no large scale domain or crystallite formation observed in our bilayers as evidenced from differential interference contrast (DIC) image (see Fig. S12, SI).

We have recently shown that incubation of binary phospholipid-cholesterol model bilayers with cholesterol dependent pore-forming proteins, Listeriolysin O (LLO), at lower cholesterol concentration (below 50%), leads to appearance of dynamically heterogeneous nanoscale proteo-lipid domains^39^. The formation of such nanoscale domains is induced by the affinity for cholesterol with the LLO protein and mediated by cholesterol reorganization in the membranes. However, is it possible that such domains can exist even in the absence of proteins driven simply by the enhanced concentration of cholesterol? In other words, is protein a necessary component in membranes for the appearance of nanoscale dynamical heterogeneity or can they emerge as a consequence of cholesterol-phospholipid interactions above a threshold cholesterol concentration in a two-component phospholipid-cholesterol bilayer.

### Spatially resolved lipid dynamics at low cholesterol concentration using STED-FCS

**Figure 2:**
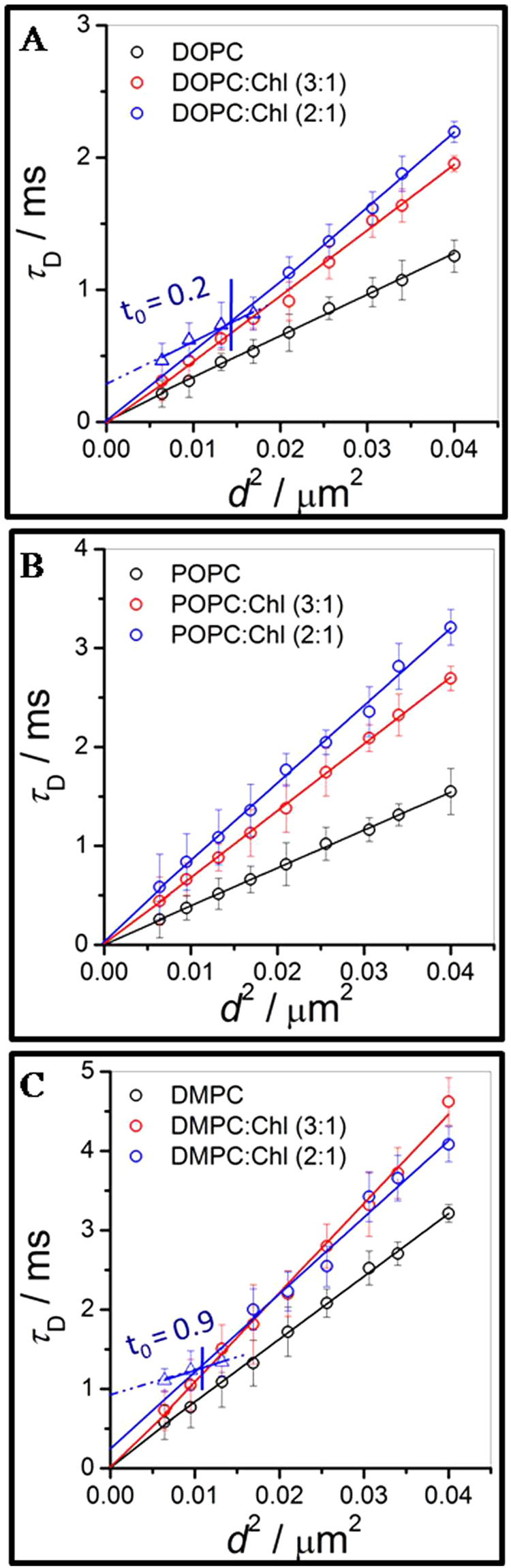
Dependence of transit time (*τ*_*D*_) on the focal spot area, *d* ^2^ - FCS diffusion law. The respective *τ*_*D*_ values were extracted from the correlation data of pristine (black), with 25% (blue) and 33% (red) cholesterol content in lipid bilayers of (**A**) DOPC, (**B**) POPC and (**C**) DMPC bilayers. The solid lines are the linear fit using Eq. (1) in the various diffusing regimes while the dotted lines represent extrapolations to highlight the nature of deviation from the expected Brownian diffusion behavior as per FCS diffusion law, in the respective regimes. The vertical lines, in respective panels, indicates the crossover length scale, *ξ*, between two dynamical regimes characterized by free or hindered lipid diffusion. The STED-FCS measurements were performed within 4–5 h of the LB transfer at 24 ± 2 °C.

For this purpose, we performed FCS in super-resolution STED mode^40^ by varying the STED excitation power to reduce the observation volume to well below the diffraction limit (<200 nm). The calibration of focal spot diameters (*d*) were made from the STED-FCS measurements of the Atto488-PE fluorescent lipid in supported pristine DOPC lipid bilayers (SI). Pristine refers to a pure lipid bilayer without cholesterol which is not expected to show any anomalous dynamics. Figure 2 describes the dependence of the transit time *τ*_*D*_ as a function of the observation diameter (*d*), as extracted from the respective STED power dependent auto-correlation data of pristine phospholipid and phospholipid-cholesterol binary mixture bilayers. The power dependent correlation data reflects the nature of diffusion of lipids in the bilayer membrane corresponding to this length scale, *d*. The variation of *τ*_*D*_ with *d* ^2^ can provide insight into the underlying diffusion mechanism in the system^54–58^. The transit time,

1 $${\tau }_{D}=\frac{{d}^{2}}{8{D}_{eff}ln2}+{t}_{0},$$

where, *d* is the diameter of the confocal or STED focal spot, *D*_*eff*_ is an effective diffusion coefficient and *t*_0_ is the intercept. For Brownian diffusion *t*_0_ = 0, and *D*_*eff*_ = *D*. The diffusion coefficient, *D*_*eff*_, can be obtained from the slope of the *τ*_*D*_ vs *d* ^2^ data and is tabulated in Table 1 using Eq. 1. Non-zero values of *t*_0_ (positive or negative) have been connected to various non-Brownian mechanisms due to hindered diffusion involving presence of fluid-like nanodomains, meshwork structure or gel-like nanodomains^59–63^. The domain size, *ω* can be estimated by setting *d* = *ω* for *τ*_*D*_ = 0 in Eq. 1. This leads to a valid domain size only when *t*_0_ < 0 from Eq. 2 ^62^,

2 $$\omega =\sqrt{8ln2{D}_{eff}|{t}_{0}|}.$$

While *t*_0_ > 0 is indicative of an underlying hindered diffusion mechanism, estimating the domain size in this regime is not straightforward. Hence we only provide domain size estimates for *t*_0_ < 0. Nevertheless, *t*_0_ < 0 could also describe meshwork-like diffusion, which is however unrealistic in a two-component system as described before^63^. For DOPC bilayers (Fig. 2A), *τ*_*D*_ increases with increase in cholesterol content for all values of *d* ^2^ reflecting the lower values of *D* with increasing cholesterol content. Up to 25% cholesterol (open red circles) Brownian lipid diffusion was observed for all length scales (*d*). In contrast, for 33% cholesterol containing DOPC bilayers (open blue circles), a dynamical crossover is observed at a length scale, *ξ* ≃ 120 nm. Although similar dynamical crossover phenomenon was observed in our earlier work^38^ with DOPC:Chl(3:1) bilayers incubated with pore forming proteins, this is a unique observation of such crossover behavior in binary DOPC-cholesterol membranes. Above *ξ*, lipid dynamics corresponds to free diffusion (*t*_0_ = 0) while below this length scale the positive value of *t*_0_ (~0.2) suggests the emergence of hindered lipid diffusion due to the presence of dynamically partitioning nanodomains^54, 59–61^. In contrast for POPC bilayers, we observed Brownian diffusion (Fig. 2B) for all cholesterol concentrations up to 33% cholesterol, for all *d*, indicating the absence of any substructures at least above the lowest length scale (80 nm) sampled in our STED-FCS experiments. For the case of pristine and 25% cholesterol containing DMPC bilayers (red open circle), the diffusion is Brownian. We note that *τ*_*D*_ values for pristine DMPC are always higher than the pristine DOPC and POPC bilayers irrespective of any STED power, indicating the lowered gel-like diffusivities (Fig. S6, SI) in DMPC when compared with the more fluid-like DOPC and POPC bilayers at the measurement temperature. However, at 33% cholesterol (open blue circle), a distinct dynamical crossover at  *﻿ξ* =103 nm was observed for the DMPC bilayer. Unlike the case of DOPC, free Brownian diffusion is not observed in either of the regimes, above and below *ξ*, and *t*_0_ values were found to be 0.26 and 0.9, respectively.

**Table 1:** Estimation of domain size from FCS diffusion law.

System	PSF	*D*_*eff*_ (μm^2^ s^−1^)		*ω* (nm)	
		(*S*)	(*F*)	(*S*)	(*F*)
DOPC:Chl(1:1)	*d* > *ξ*	1.14	2.96	119 ± 4	—
	*d* < *ξ*	3.19	4.29	—	—
POPC:Chl(1:1)	*d* > *ξ*	1.36	1.84	—	106 ± 5
	*d* < *ξ*	1.97	8.51	—	—
DMPC:Chl(1:1)	*d* > *ξ*	0.96	2.74	97 ± 4	—
	*d* < *ξ*	3.29	6.29	—	—

### Unravelling intra-domain lipid dynamics at high cholesterol concentration

**Figure 3:**
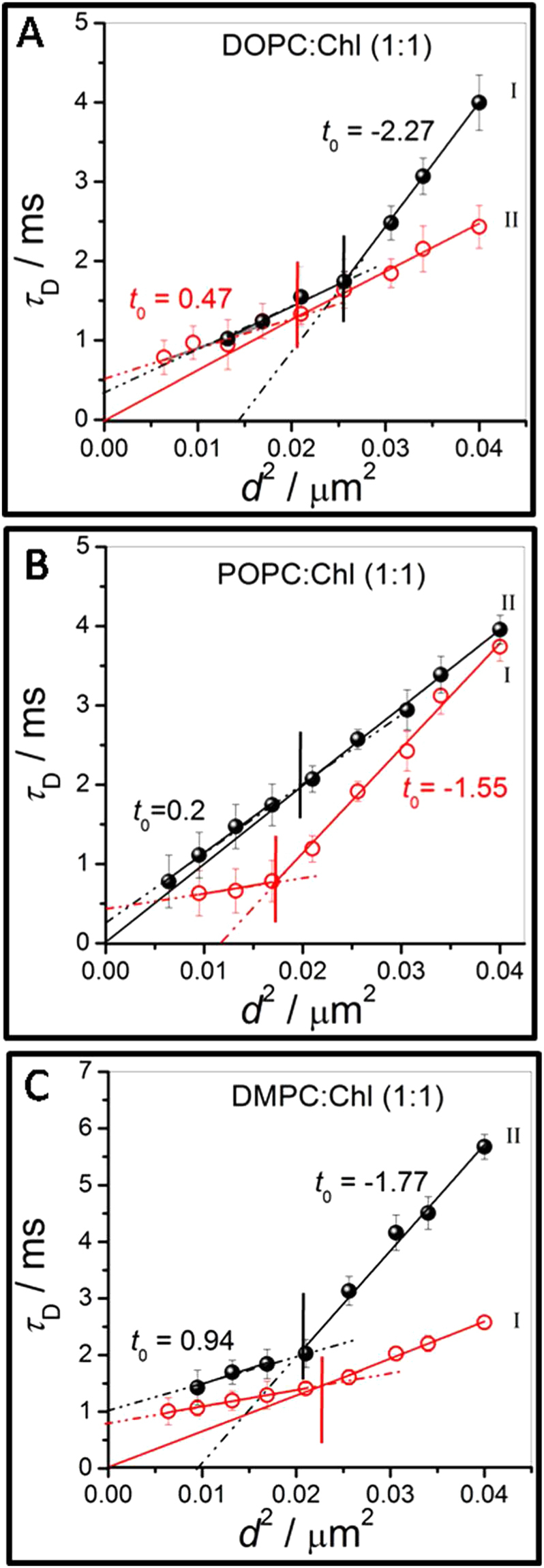
(**A**–**C**) represents FCS diffusion law plots for DOPC, POPC and DMPC bilayers respectively. Each of the bilayers are embedded with 50% cholesterol. The closed and open symbols represent data corresponding to slow (*S*) and fast (*F*) diffusivity. The diffusivity data corresponding to the spatially resolved points (see Fig. 1) are marked as ‘I’ and ‘II’. The vertical lines, in respective panels, demarcates the crossover length scale, *ξ*, separating different dynamical regime observed in each of these regions I and II, which have also been identified as either *S* or *F* (refer main text for details) depending on the mean lipid *D* values in these regions. All the STED-FCS measurements were performed within 4–5 h of the LB transfer at 24 ± 2 °C.

At 50% cholesterol concentration (Fig. 3A–C), we observe two dynamically distinct regions referred to as *S* and *F* (Fig. 1) for all the lipids, with the distinction being the strongest for DOPC and the least for POPC. STED-FCS allows us to compare and differentiate between the lipid dynamics in these dynamically distinct regions which manifest at length scales of 100 nm or less. The length scale dependent transit time data for DOPC (cf. Fig. 3A, closed symbol) in the domain I (*S*) reveals a distinct crossover at, *ξ* = 160 nm above which the intercept is negative (*t*_0_ = −2.27) while for *d* ^2^ values below *ξ* a positive intercept (*t*_0_ = 0.29) is observed. Thus in both regimes Brownian diffusion is not observed. The length scale at which dynamical heterogeneity, as revealed by the crossover, is clearly enhanced when compared with the 33% DOPC cholesterol bilayer. The very large negative intercept which has been observed earlier in lipid bilayer membranes has usually been associated with hindered lipid dynamics in the presence of a meshwork^54, 59–61^. However this situation has also been attributed to dynamics due to gel-like domains^62, 63^ which is the more relevant interpretation for the supported bilayer platforms used in this study. These observations are quite unique since both dynamical regimes show hindered diffusion. In contrast for region II (*F*), which from our images appear to represent the majority fraction (Fig. 1), the overall dynamics is closer to Brownian with a weak crossover observed at 142 nm, which is smaller than the crossover observed for region *S*. Significantly the STED-FCS data reveals for the first time distinct intra-domain lipid dynamics with strong heterogeneity in the cholesterol enriched region *S* and expectedly, more uniform and fluid-like dynamics in the region *F*, due to nanodomain partitioning. STED-FCS thus reveals this unique nanodomain dynamical texture within dynamically distinct regions, in an otherwise fluid phospholipid bilayer, which has not been observed thus far with various strategies for spot variation FCS.

Contrast this behavior with that observed for POPC:Chl (1:1) bilayers shown in Fig. 3B. Consistent with the confocal FCS observations of a small difference in mean lipid *D* (Fig. 1K) the *τ*_*d*_ values in region I (*F*) and II (*S*) are similar at larger *d* ^2^. However significant differences emerge upon reducing *d* ^2^. More interestingly, region II (*S*) shows almost free Brownian diffusion over the entire range of observable *d* ^2^, in our experiments, whereas region I (*F*) shows evidence of hindered diffusion and heterogeneous dynamics with a strong crossover at *ξ* = 127 nm. Further, *t*_0_ is negative, suggestive of lipid dynamics in gel-like domains, above *ξ* = 127 nm, while it exhibits a small but finite positive value for *t*_0_ below this length scale which is indicative of small deviation from free diffusion due to dynamically partitioning domains^60, 62^. In contrast in region *S* only a weak dynamical crossover is observed at *ξ* = 148 nm. This is also opposite to what was observed for DOPC bilayers.

For DMPC bilayers, on the other hand, Fig. 3C shows that both regions I (*F*) and II (*S*) exhibits distinct dynamical crossovers. In region *F*, Brownian diffusion is observed above *ξ* ≃ 150 nm and shows nanodomain mediated hindered diffusion below this length scale. In region *S*, a strong dynamical crossover at *ξ* = 142 nm separates regions having negative intercept of *t*_0_ = −1.77, corresponding to lipid dynamics in gel-like regions (*ω* ~ 97 nm) followed by dynamics in more permeable nanodomains below this length scale *ξ*. The various parameters obtained from analysis of the STED-FCS data, for the 50% cholesterol containing lipid bilayers is summarized in Table 1. In cases where *t*_0_ > 0 it is not possible to extract domain sizes, *ω*, either using Eq. 2 or using the dynamical crossover length scale, to free diffusion, for smaller values of *d* ^2^. What is significant is the unique observation of dynamical crossovers at length scales ranging from 100–150 nm which is enabled by our STED-FCS measurements including cases where the dynamics does not correspond to free diffusion in either regime. To our knowledge such dynamical anomalies have not been observed earlier with any mode of FCS measurements.

In order to check whether these observed dynamical heterogeneities are caused by pinning of lipids due to inherent substrate effects we carried out similar measurements (cf. Figs S13–16, SI) on polymer cushioned bilayer platforms which have been widely used in the supported bilayer literature^64, 65^. In our study, we have used poly(acrylic acid) (PAA) which is routinely used as a polymer cushion. In the polymer cushioned bilayers, we observed qualitatively similar morphology and lipid dynamics with distinct crossover phenomena (cf. Fig. S16, SI) as reported for the supported bilayers prepared on uncushioned substrates.

## Discussion

In this work, we studied cholesterol concentration dependent nanoscale heterogeneity directed by the interplay of cholesterol concentration and phospholipid properties in minimal two-component phospholipid-cholesterol bilayers. Our STED-FCS results below the diffraction limit (<200 nm) provide crucial insights toward the length scale dependent diffusion crossover due to the presence of substructures at higher cholesterol content. Domain formation as visualized by confocal microscopy images are pronounced for symmetric DOPC and DMPC lipids when compared with the asymmetric POPC lipid. POPC is known^46^ to partition cholesterol to a lesser extent due to the presence of single saturated tail^42^. As a consequence cholesterol organization and domain formation is reduced with POPC when compared to the symmetric lipids, DOPC and DMPC. While we do not study dynamics of a “single” nanodomain (of dimension ~100 nm or less) when we sample lipid dynamics within a macrodomain of dimensions ~200–600 nm or more, in the absence of heterogeneity one would expect the diffusion to be Brownian at all length scales. However, depending on cholesterol and lipid composition we indeed find different values of lipid diffusivities at different length scales indicating inherent heterogeneity of lipid dynamics driven by the local environment/viscosity etc. Recent MD simulations^33^ of single component DPPC membranes suggests that dynamic heterogeneity and nanodomain formation can be driven by density correlations akin to supercooled liquids, and can occur in the absence of critical fluctuations or inherent compositional heterogeneity commonly associated as a requirement for domain formation and dynamic heterogeneity. Significantly, there are no reports of nanoscale domain formation or dynamic heterogeneity in fluid phase or liquid crystalline bilayers.

Using STED-FCS we establish the conditions for onset of nanodomains with clearly quantified length scales below the optical diffraction limit in simple cholesterol-phospholipid bilayers. The emergence of nanoscale heterogeneity and the length scales at which this occurs is a strong function of the cholesterol content. At 25% cholesterol we do not detect the presence of dynamic heterogeneity in any of the lipid bilayers investigated. However at 33% we observe the emergence of weak nanoscale dynamical heterogeneity only in STED-FCS mode for DOPC and DMPC bilayers, but not for POPC. Hence the measured dynamical crossover length scales, *ξ*, for DOPC and DMPC, provides direct evidence for the existence of such dynamical nanodomains in bilayer membranes even in a situation where there is no manifestation of macro-phase separation. Thus, the STED-FCS data at the lower cholesterol concentrations already provides crucial insights about the existence of inherent nanoscale heterogeneity in the lipid dynamics and their dependence on cholesterol-phospholipid interactions^1, 14, 32^. It is possible that heterogeneity, if present in the case of POPC, might occur at length scales below our instrument resolution of 80 nm or at higher cholesterol concentration. At 33% cholesterol and measured temperatures, DMPC, which is the only high melting lipid investigated in this study, lies in the proximity of *L*_*o*_/*L*_*d*_ phase boundary^43, 66, 67^. Although clear phase separation is not observed in these bilayers, the observed dynamical heterogeneity is possibly connected with this location on the phase diagram. In the case of DOPC where there is no phase co-existence^68, 69^ the weak nanoscale heterogeneity suggests the formation of nanodomains driven by non-ideal mixing of disordered low melting phospholipids and cholesterol^17, 44, 67, 70, 71^.

At higher cholesterol concentrations (50%) DOPC and DMPC are expected to lie in the *L*_*o*_ part of the phase diagram^48^. For these bilayers, cholesterol rich and poor domains can be discerned with length scale of 200–600 nm in confocal microscopy as indicated earlier. Lipid saturation is found to play a critical role, with cholesterol rich domains showing slower diffusivity in the DOPC bilayers while cholesterol rich domains in DMPC show an opposite trend with higher diffusivity, compared to their complementary phase in their respective bilayers. The formation of cholesterol rich and poor domains, and their respective nanoscale intra-domain lipid dynamics illustrate the interplay between phospholipid-cholesterol interactions and phospholipid type; stronger interactions with DMPC which leads to greater fluidization in cholesterol rich regions (F) and weaker interactions with DOPC resulting in lower mobility in cholesterol rich regions (S). In POPC even at 50% cholesterol concentration macroscale domain formation is not expected and this is consistent with the absence of regions with well separated lipid mobilities in confocal FCS as observed for DOPC and DMPC. However in STED-FCS we not only find strong evidence of the presence of two populations of lipid mobilities as the probe volume is reduced below the diffraction limit, we also observe significantly different dynamics in these two populations.

Our study thus reveals that the extent of heterogeneity above an optimal cholesterol concentration is strongly influenced by the physical properties of phospholipid component which dictates lipid re-organisation and microstructure at the nanoscale. Various reports suggest that the role of solid substrates and bilayer preparation methods influence the complex dynamics and phase behavior in bilayer membranes^72–77^. However our results indicates that the observed morphological and dynamical heterogeneity as evidenced from the crossover in diffusion behavior are intrinsic to the lipid bilayer composition and not a function of the underlying substrate (polymer or glass). The absence of direct contact either in uncushioned (presence of ~2–3 nm water layer) or polymer cushioned bilayers, helps mitigate substrate effects for the diffusion measurements. On the contrary, it is worth mentioning that substrate-bilayer interactions may have some similarity to the coupling between the plasma membrane and cytoskeleton matrix in real cells, lending additional support to the use of solid supported bilayers in our study.

While it is widely believed that appearance of *L*_*o*_ nanodomains in an *L*_*d*_ environment requires the presence of both low and high melting lipids along with cholesterol which is expected to lead to the co-existence of fluid phases, our results establish that neither of these conditions are absolutely necessary for the formation of such domains. In three-component membranes, one would expect a fast diffusion in a homogeneous *L*_*d*_ membrane and slow diffusion within a homogeneous *L*_*o*_ phase. However in our case, the dynamic heterogeneity (crossover in diffusion behavior) which occurs in both the homogeneous membrane as observed at intermediate cholesterol compositions (33%) and within a homogeneous region (cholesterol rich and poor domains at 50% cholesterol containing membranes) suggests that these are not limited to domain formation. On the other hand, the fact that nanoscale dynamic heterogeneity and domain formation is not connected to phase separation indicates that the origins of the dynamical crossovers could be similar to the dynamic heterogeneity related to caging effects commonly observed in supercooled liquids and glasses^78, 79^. Further the emergence of these nanoscale heterogeneities could also be attributed to non-ideal lipid mixing^67, 80^ leading to formation of regions which are otherwise difficult to detect by conventional microscopy methods. In addition, in an alternate model based on a microemulsion perspective^81, 82^, size estimates of these nanodomains are ~100 nm, which is remarkably similar to the observed values of domains sizes and crossover length scales in our STED-FCS measurements.

## Conclusions

In summary, our results reveal the existence of a rich level of nanoscale dynamic heterogeneity even in minimal systems, consisting of two- component phospholipid-cholesterol bilayers. Our STED-FCS results provide, for the first time, direct estimates of the length scale of dynamical nanoscale domains as well as the inherent heterogeneity that exists within these domains themselves. Further, the complexity of nanodomain formation and dynamics is revealed by crossovers between regimes ranging from free to hindered or even between two types of hindered diffusion. Extent of cholesterol partitioning and type of bulk phospholipid properties determines this intra-domain dynamical heterogeneity indicating their intimate correlation. The key results of our work lies in the observation of Brownian and non-Brownian lipid dynamics within these “substructures” in otherwise homogeneous spatial regimes. This non-Brownian dynamics is manifested both in terms of the observation of a non-zero intercept in FCS diffusion law plots, below the diffraction limit, signifying hindered lipid diffusion, as well as clear dynamical crossovers within each of these domains. While the FCS diffusion law anomalies has been attributed to regions of different lipid mobilities, compared to the surrounding, the observation of dynamical crossovers can be interpreted to represent a length scale which separates two regions of distinct lipid dynamics. Further, the observed dynamic heterogeneity observed in this study is independent of the substrate and depends solely on the bulk membrane composition and chemistry. Understanding the underlying dynamics below the diffraction limit in minimal two-component model biomembrane platforms especially at high cholesterol content, will shed light on the sub-structures associated with functional nanodomains present in the real cell membrane. Finally, with the advent of super-resolution gated STED^83, 84^, where resolutions up to 30 nm are feasible, assessing the complexity due to surface curvature, presence of transmembrane proteins and cytoskeleton effects on the dynamics and assembly of plasma membrane components at the nanoscale will continue to provide novel insights in membrane biology and biophysics.

## Materials and Methods

Phospholipids such as DOPC (>99% purity), POPC (>99% purity) and DMPC (>99% purity) and cholesterol (Chl, >98% purity) was obtained from Avanti polar lipids. 3-aminopropyl triethoxysilane (APTES), and 450k MW poly(acrylic acid) (PAA), were purchased from Sigma-Aldrich (USA) and used without further purification. Fluorescent probe 1,2-dimyristoyl-*sn*-glycero-3-phosphoethanolamine labeled with Atto488 was obtained from ATTO-TEC GmbH, Germany. The lipid solutions (1 mM) were prepared in chloroform (HPLC grade, Sigma Aldrich) and sprayed on the water subphase for the formation of interfacial monolayers. Ultrapure water with a resistivity of 18.2 Ωcm was used as the subphase for all monolayer studies produced by a two-stage Elix-3 and Milli-Q (Millipore Academic) system. Prior to the experiment, the mini trough was cleaned with ethanol (extra pure AR grade, Fine Chemicals, India) several times and finally rinsed with ultra pure water.

### Fabrication of supported lipid bilayers

Supported lipid bilayers are prepared by the layer-by-layer transfer (two layers) of lipid monolayers onto a hydrophilic glass (Piranha treated) substrate (20 × 20 mm, Glaswarenfabrik Karl Hecht GmbH and Co KG, Germany); this planar configuration is ideally suited for observation using fluorescence microscopy. To make the bilayer luminescent, dye tagged lipid (Atto488-PE, 5 × 10^−4^ mol%) was mixed thoroughly with pristine phospholipid or phospholipid-cholesterol mixture before spreading at air-water interface. Multiple compression-expansion cycles of the monolayers are followed at a constant trough temperature of 15 ± 1 °C before the collapse surface pressure. The layer-by-layer transfer of monolayers were transferred at a highly condensed surface pressure of 35 mNm^−1^. PAA cushioned bilayers were prepared by using the procedure adopted by El-khouri *et al*.^85^. In brief, the Piranha treated glass substrates were transferred to a toluene solution of (aminopropyl)triethoxysilane (APTES) with APTES/toluene = 1:50(vol/vol) and were kept at 100 °C for 2 h. The unbound APTES was removed by gently washing with toluene. PAA solution (3 mg/ml) in methanol was then spin coated (2000 rpm, 30 s) and kept for 4 h at 200 °C which induced amide formation between the PAA carboxylic acid and the amine functionality at the surface APTES. The two leaflets of the bilayers were deposited on this PAA cushion layer by using Langmuir-Blodgett/Langmuir-Schaefer (LB/LS) technique. After transfer, the bilayers were always stored under water without exposing to air for further use.

### STED-FCS measurement and analysis

For imaging and FCS, we applied STED-FCS nanoscopy using a commercial CW-STED setup (SP5x, Leica Microsystems GmbH, Mannheim, Germany). FCS data acquisition was set for a total duration of 10 s for both confocal- and STED-FCS recordings. The detailed descriptions of analysis are given in SI. At least 30 to 50 independent measurements were made for each sample at different positions and the FCS protocol was repeated for three independent sample sets to confirm the reproducibility of the data.

## Electronic supplementary material

Supplementary Information
