# Supplementary material for: Complex dynamics at the nanoscale in simple biomembranes
Source: Sci Rep. 2017 Sep 11;7:11173. doi: 10.1038/s41598-017-11068-5 (PMC5593986; doi:10.1038/s41598-017-11068-5)
Supplement: Supplementary file 1 — Supplementary Information [file 41598_2017_11068_MOESM1_ESM.pdf]

Supporting information

Complex dynamics at the nanoscale in simple biomembranes

Nirod Kumar Sarangi[1], K. G. Ayappa[2,3*], and Jaydeep Kumar Basu[1*]

[1]Department of Physics, [2]Department of Chemical Engineering, [3]Center for Biosystems Science and Engineering, Indian Institute of Science, Bangalore--560 012, India

Table of Contents

Table of Contents	Page No
**Pressure-area isotherms and layer-by-layer phospholipid monolayer transfer for fabrication of SLBs**	**2-4**
**STED-FCS Nanoscope**	**4-9**
**Spatio-temporal lipid dynamics upon β-cyclodextrin treatment**	**10-15**
**Lipid dynamics on polymer cushioned bilayer**	**16-19**

Pressure-area isotherms and layer-by-layer phospholipid monolayer transfer for fabrication of SLBs: Surface pressure versus area-per-molecule isotherms were recorded using the KSV LB rectangular mini trough (area, 240 cm^2) equipped with a Wilhelmy balance. A platinum sensor of accuracy 0.1 mNm^{-1} was used to measure the interfacial surface pressure. A chloroform solution of either one component DOPC, POPC and DMPC lipid or binary mixture containing varied cholesterol content such as 3:1, 2:1 and 1:1 were spread on the air–water interface of a LB trough at 15±1 ºC using a precise Hamilton syringe to make a compact monolayer. After evaporation of chloroform, the isotherms were recorded at constant temperature and a barrier speed of 5 mm/min.

Figure S1 shows the pressure-area isotherm of pristine DOPC, POPC and DMPC monolayer and with varied cholesterol composition starting from 3:1, 2:1 and 1:1. The mean molecular area of DOPC, POPC and DMPC are ~85, 73 and 55 $Å^2$/molecule respectively. The decrease in mean molecular area is due to the decrease in hydration of the head group and higher van der Waals interaction among the alkyl chains. Upon addition of 25% cholesterol in the respective monolayer, the mean molecular area further decreases complying with the condensing effect of cholesterol. At higher percentages of cholesterol such as 33 and 50%, the mean molecular area increases and no demixing behavior was observed (note, the step-like feature observed for pure cholesterol (Fig. S1a, green) is absent in mixed lipid) even at the highest cholesterol concentration used. This provides partial evidence that cholesterol precipitation does not occur at these concentrations. The condensing effect of cholesterol is due to the intermolecular cooperative interaction between the cholesterol –OH group and lipid head group and degree of rigidity of the monolayer. Our results also further supported by the maximum compressibility $\beta = -\frac{1}{A}\frac{\delta A}{\delta \pi}$ data as shown Figs. S1b, d and f.

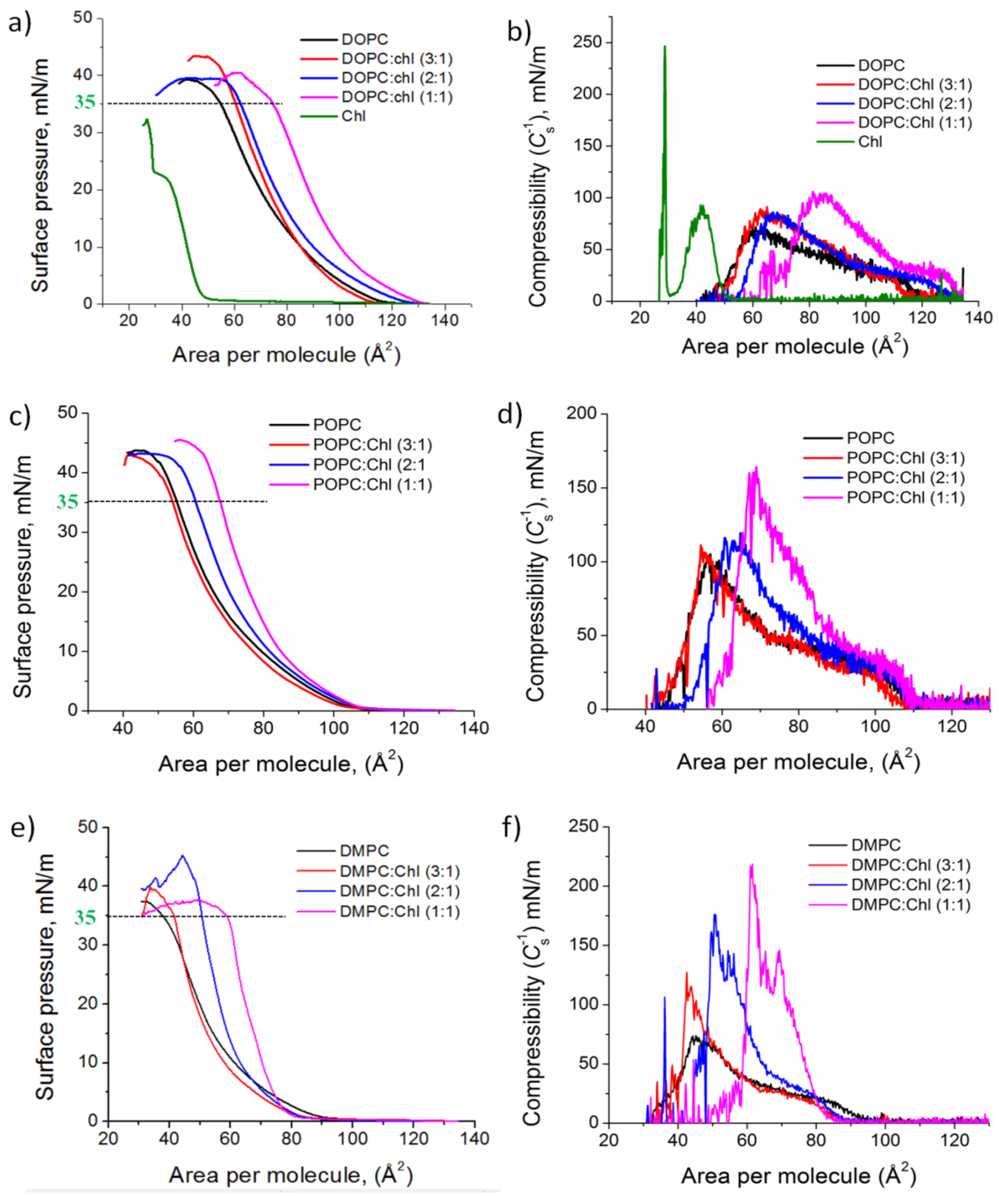

Figure S1. (a-c) illustrate surface pressure-area isotherms of pristine DOPC, POPC and DMPC respectively and in each panel, lipid to cholesterol ratios are 3:1, 2:1 and 1:1. The isotherms and the LB transfer of binary phospholipid-cholesterol mixtures were carried out at 15±1 ºC. Supported lipid bilayers were prepared at a highly condensed surface pressure of 35 mN/m as marked in the horizontal dotted line. Right panels show the compressibility plots of the respective monolayers derived from the π–A data.

Supported lipid bilayers of single- or two-component lipids were formed upon controlled transfer of lipid interfacial monolayers onto pre-treated glass substrates by employing the

Langmuir-Blodgett (LB) method. During the preparation of bilayer using the LB method, multiple compression-expansion cycles were followed before the collapse surface pressure and subsequently the bilayers were transferred at a highly condensed surface pressure of 35 mNm^{-1} to the hydrophilized glass slides by using layer-by-layer transfer. Prior to transfer, glass substrates (20mm×20mm, Germany) were cleaned using "piranha solution" (a 30:70 mixture of 30% hydrogen peroxide and concentrated sulfuric acid at 80 ºC) for 30 min and washed multiple times with MilliQ DI water (resistivity ~ 18.2 MΩ.cm). The first monolayer was transferred at an equilibrium pressure of 35 mN/m by vertical withdrawal of the substrate at a speed of 5 mm/min with a transfer ratio of ≤ 1.2±0.1. The second monolayer transferred at the same surface pressure by a vertical down stroke yielded a centro-symmetric bilayer (Y-type) on the support. To make the bilayer luminescent, dye tagged lipid (Atto488-PE, 5×10^{-4} mol%) was mixed thoroughly with pristine phospholipids or phospholipid-cholesterol mixtures before spreading at the air-water interface. After transfer, the bilayers were transferred to a container under water and stored at 25ºC for further use. All measurements were done on the prepared bilayers within 4-5 h of the LB transfer at 24±2 ºC.

STED-FCS Nanoscope

For imaging and FCS, we applied STED-FCS nanoscopy using a commercial STED setup (SP5x, Leica Microsystems GmbH, Mannheim, Germany). Ar ion laser and a STED laser (592 nm) with a continuous-wave (CW) mode were aligned in such a way to accomplish a doughnut-shaped focal intensity distribution featuring a central intensity zero and diffraction-unlimited spot. The master power of the Ar laser was set to 25-30% and subsequently excitation at 488 nm was used at 1–25% output power. The CW-STED 592 nm laser was operated at 0–100% output power (varying in the range 0–260 mW measured directly at the focal plane of a 10x air objective). Before each series of measurements, the auto-alignment procedure (super-imposing the excitation laser and the depletion lasers) was performed in 25nm×25nm pixel area. This procedure was repeated every 15 minutes. An oil immersion objective, 100x 1.4 NA envisage focusing of the superimposed excitation and STED laser beams as well as collection of the fluorescence emitted intensity. The emitted intensity was guided to the microscope objective back aperture through the confocal pinhole (set to 1 Airy unit) filtered by a 594 nm notch filter imaged onto a single-photon-counting avalanche photo-diode (APD; Micro Photon Devices,

PicoQuant, Berlin, Germany) in the external port of the microscope with a band-pass filter (BS 560) between 500–550 nm. All the images as shown in Fig. S2-4 are 512 × 512 pixels; line average was set at 2 and scan speed at 600 Hz.

Analyses of FCS:

The particle number fluctuations $N(t) = \langle N \rangle + \delta N(t)$ of fluorescing molecules entering and leaving the focus of a confocal microscope during the excitation could be calculated by measuring the emitted intensity $I(t)$ as $I(t) = \langle I \rangle + \delta I(t)$; where $\langle I \rangle$ and $\delta I(t)$ represents a constant offset and the fluctuations respectively. In FCS, the autocorrelation function $G(t_c)$ from the intensity signal $I(t)$ measured in the microscope was calculated using

$$G(t_c) = \frac{\langle \delta I(t) \delta I(t+t_c) \rangle_t}{\langle I(t) \rangle_t^2} \qquad \text{(S1)}$$

where $\langle \dots \rangle_t$, denotes a time average over the time variable *t*. The correlation curves were fit by Origin 8.5.0 (OriginLab) using two-dimensional one-component diffusion model assuming a Gaussian-shaped fluorescence detection profile,

$$G(t_c) = \left(\frac{1}{N}\right) \frac{1}{1+(t_c/\tau_D)^{\alpha}} \qquad \text{(S2)}$$

where,

$$\tau_D = \frac{d^2}{8Dln2} \qquad \text{(S3)}$$

τ_D denotes the average transit time through the focal spot diameter (or FWHM) d, D represents the diffusion coefficient and α the anomaly coefficient. Note that in Eqn. S2, exponent α is a fit parameter. For all the studied bilayer systems, α was found to be ~1±0.08 (see Fig. S5).

The values of d in confocal mode used throughout our calculation for estimating D were found to be ~200 nm (at 100x, oil immersion objective) and was obtained from PSF by scanning fluorescent chromeo 488 beads of 40 nm size. For calibration of the diameter of the effective focal spot (dP_{STED}) created by different STED power (P_{STED}), we performed STED-FCS measurements of Atto488 DPPE (PE) fluorescent analogues stained in both leaflets of pristine DOPC supported lipid bilayers prepared by the LB method. At STED power zero (or in normal confocal mode), the $d(P_{STED} = 0)$ and the other effective diameters at different STED power $d(P_{STED} \neq 0)$ can be obtained by using equation S4;

$$\frac{d(P_{STED}=0)}{d(P_{STED})} = \sqrt{\frac{\tau_{D(P_{STED}=0)}}{\tau_{D(P_{STED})}}} \quad \text{(S4)}$$

where τ_D is the transit times corresponding to each STED power. In the above equation, we assume that the lipid diffusion in pristine DOPC SLB undergoes two-dimensional Brownian free diffusion and the diffusion time scales proportionally with the diffusion area.

Confocal microscopy images of single- and two-component bilayers at low cholesterol content:

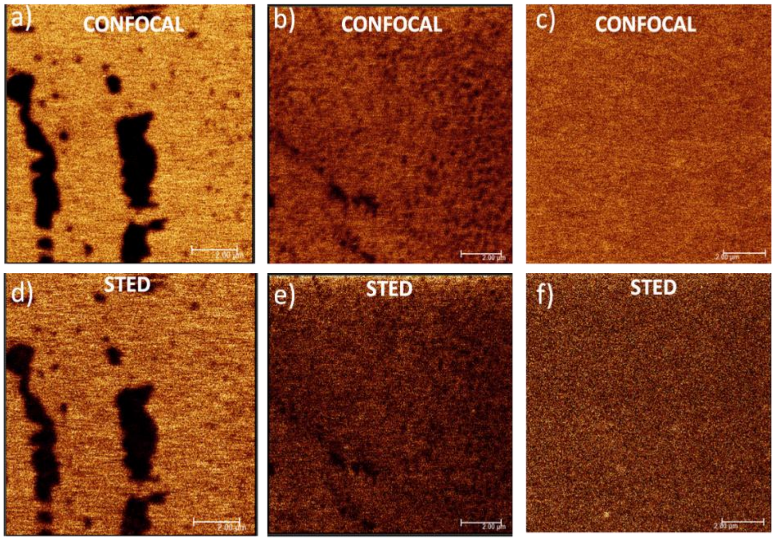

Figure S2. (a-c) Illustrates confocal microscopy images of pristine DOPC, POPC and DMPC bilayers respectively prepared using LB method. (d-f) are the respective STED microscopy images collected at same region with spatial resolution of ~80 nm. The scale bar is 2 µm.

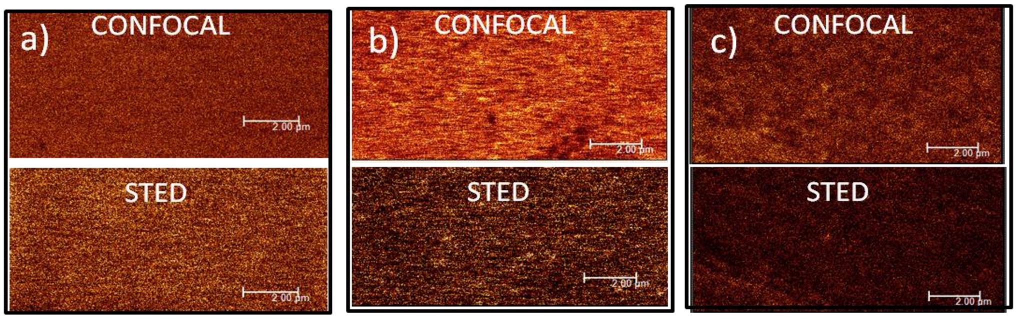

Figure S3. Top and bottom panel shows the confocal and STED microscopy image of (a) DOPC:Chl (3:1), (b) POPC:Chl (3:1) and (c) DMPC:Chl (3:1) bilayer membranes prepared by LB method. The scale bar is 2 µm.

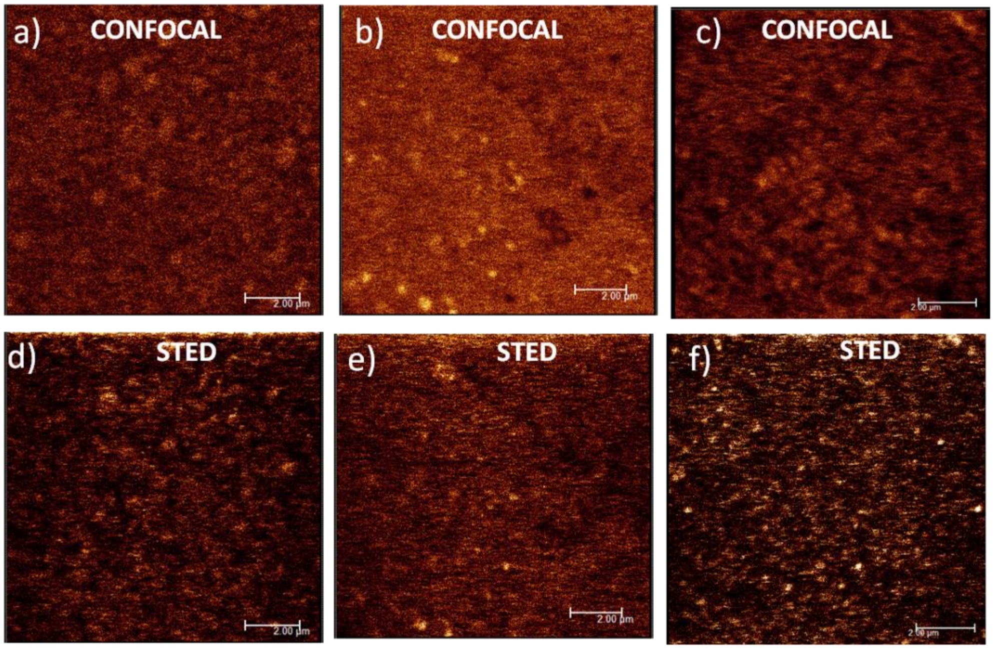

Figure S4. Confocal microscopy images of (a) DOPC:Chl (2:1), (b) POPC:Chl (2:1) and (c) DMPC:Chl (2:1) bilayers . (d), (e) and (f) are the respective STED microscopy images at ~ 80 nm spatial resolution. The scale bar is 2 μm.

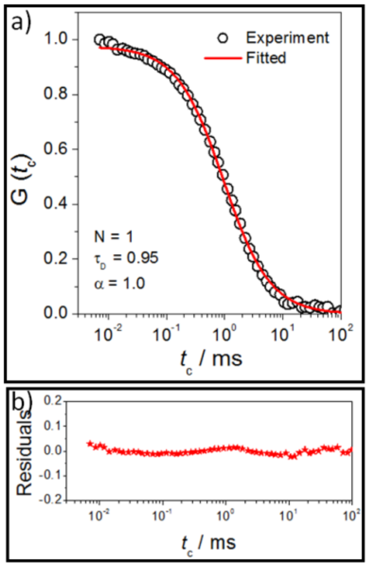

Figure S5. (a) Normalized FCS auto correlation data of DOPC bilayer recorded in confocal mode. Open symbols are the experimental data and solid lines are the fit using Eq. S2. Note, exponent α is a fit parameter. Inset shows the values for fitted parameters. (b) Illustrates residuals of the anomalous diffusion model fit, showing the accuracy of the fit.

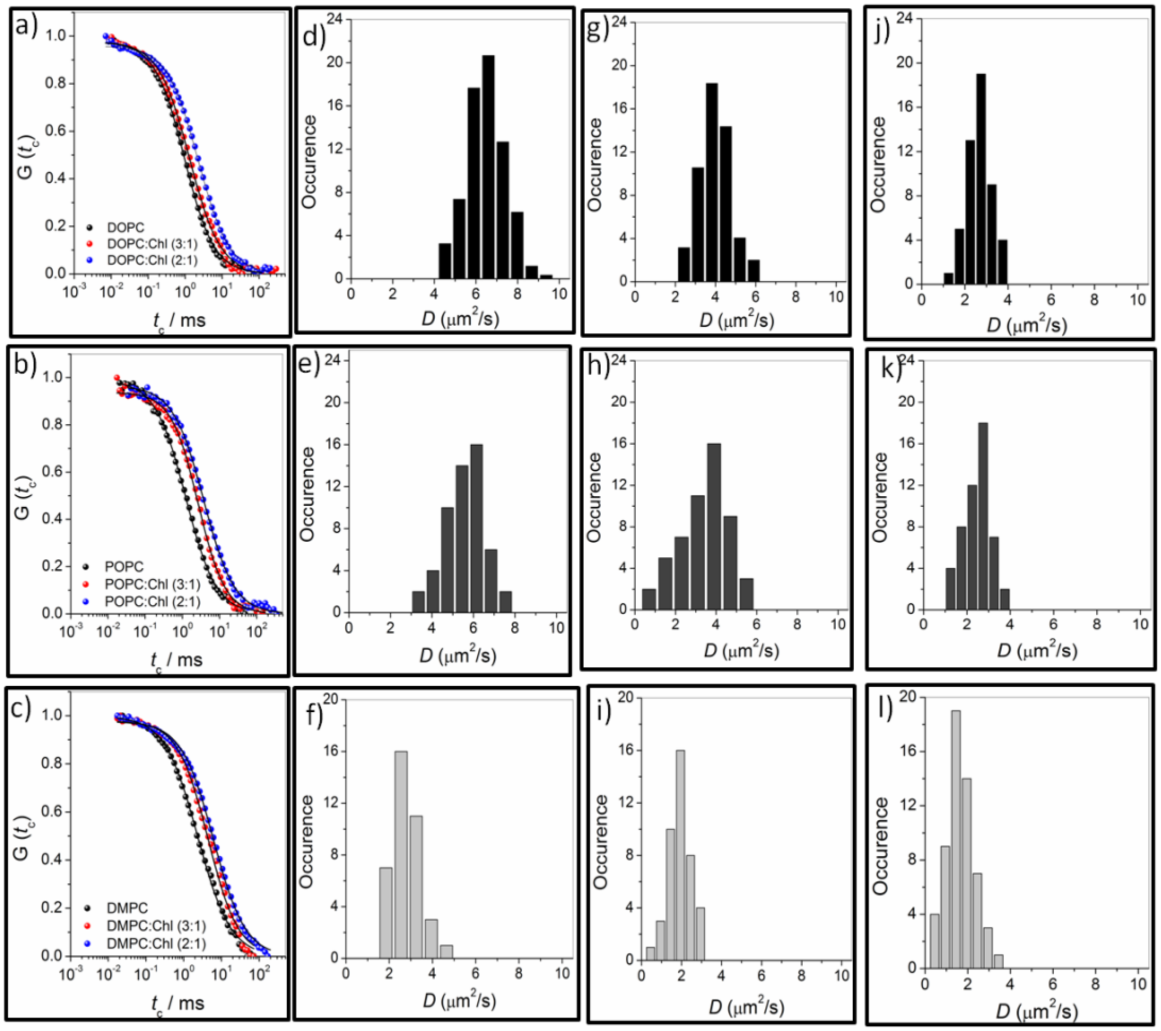

Figure S6. Representative FCS auto-correlation curves for (a) DOPC, (b) POPC and (c) DMPC bilayer in the absence and presence of cholesterol. In each panel, cholesterol free bilayer is represented by black circles, and red and blue circles are the representative correlation data for 25% and 33% cholesterol containing bilayers respectively. The autocorrelation function (G(t_c)) is plotted as a function of lag time (t_c) and the autocorrelation curve is fitted with non-linear least square fit method using equation 1 (see main manuscript). Panel d-f, g-i and j-l are the respective histograms of diffusion coefficient (D) measured from pristine lipids, 25% and 33% cholesterol containing bilayers respectively. The diffusivity data are collected from ~30-50 measurements from three independent samples.

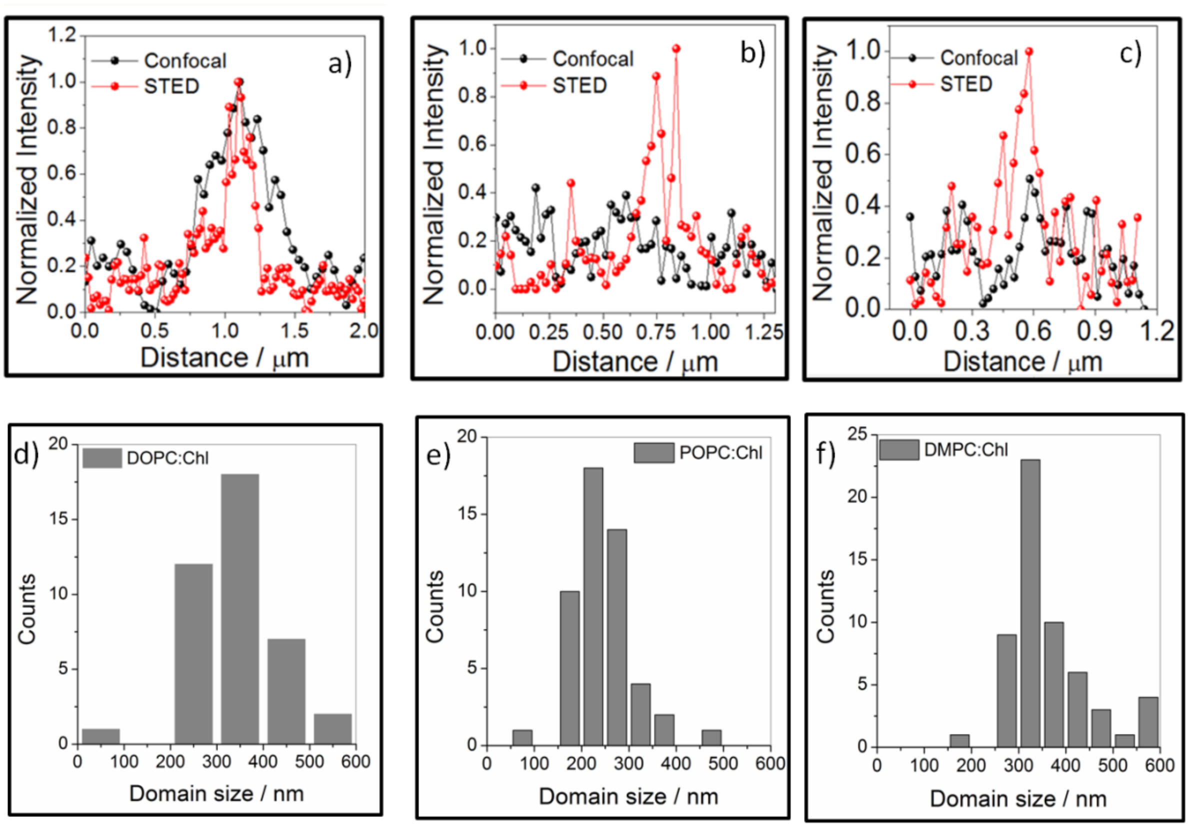

Figure S7. (a), (b) and (c) represents the intensity line profile in the region of interest (ROI) as marked in solid lines in the respective images of confocal and STED images in Figure 1 of the main manuscript. Note that the improvement of resolution as evidenced from decreasing FWHM as well as the evidence of nanosized domains can be discerned by STED but are not observed in confocal microscopy. Illustrate frequency of domain size distribution of (d) DOPC:Chl (1:1), (b) POPC:Chl (1:1) and (c) DMPC:Chl (1:1) bilayer in STED (~260 mW) mode.

Spatio-temporal lipid dynamics upon β-cyclodextrin treatment:

We explore the role of cholesterol in regions *S* and *F* (see Fig.1 in main manuscript) and their differences between different lipid types using cholesterol extraction experiment with *β*-cyclodextrin (CD). The bilayer was exposed to 2.5 mM solution of *β*-cyclodextrin in milli-Q water and the simultaneous fluorescence intensity as well as the FCS traces in confocal mode was monitored with respect to the time. Figure S8 illustrate the changes in the DMPC:Chl (1:1)

supported bilayer over the course of the CD exposure experiment. Panel b shows the intensity line profile in the region of interest (ROI) before and after 40 min of β-CD exposure time.

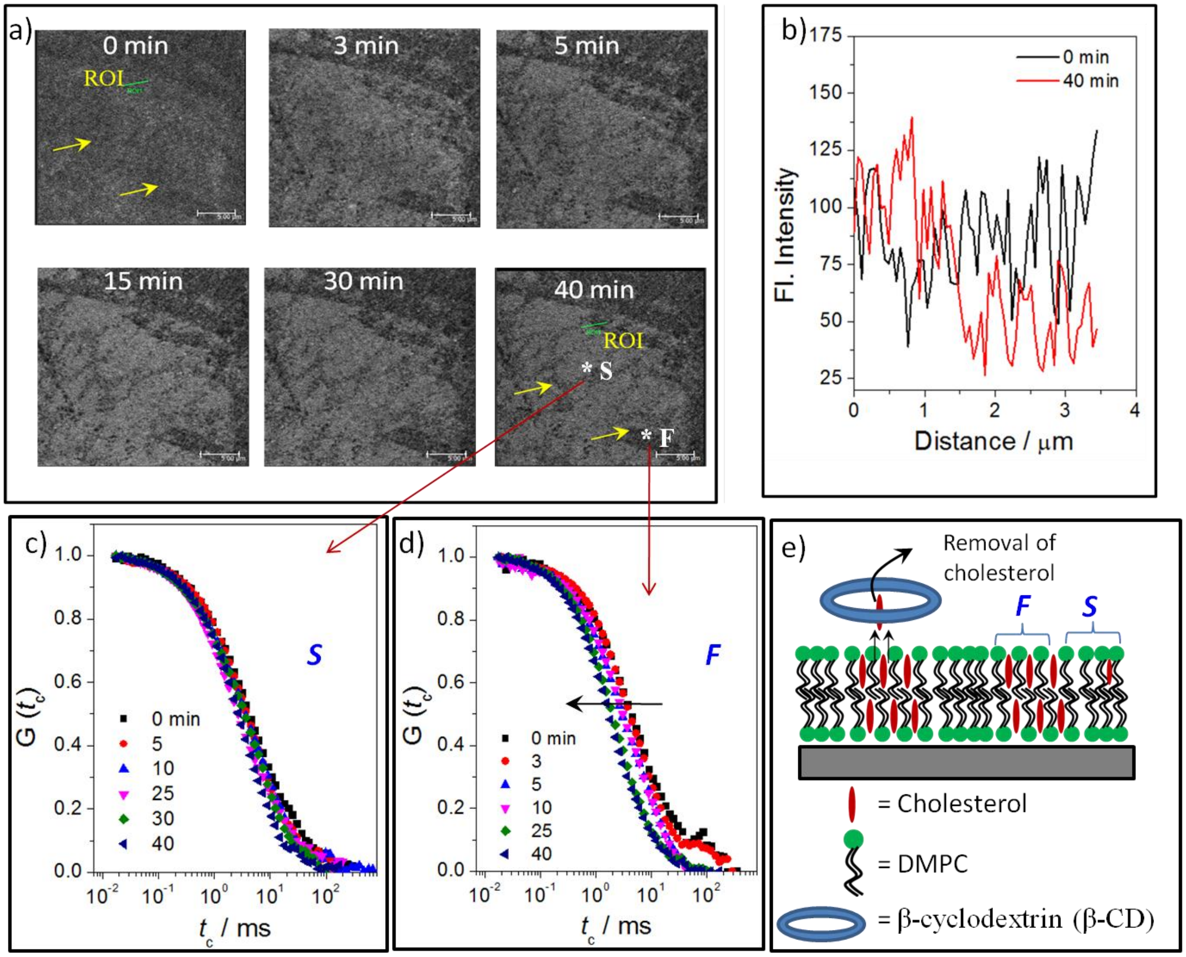

Figure S8. (a)Temporal evolution of confocal microscopy images of DMPC:Chl (1:1) upon β-cyclodextrin treatment. The decrease in fluorescence intensity in the *F* region as marked in arrow due to cholesterol depletion. (b) The intensity drops (see ROI, green line) was maximal after 30 mins (~ 50%) and remained constant for 2 hours. (c) and (d) represent the temporal evolution of FCS correlation data accrued from *S* and *F* regions respectively, showing the removal of cholesterol is more prominent from the F regions. (e) Schematic representation of cholesterol removal from cholesterol poor and cholesterol rich regions upon β-CD treatment.

The intensity drop at spatial position is suggestive of differential removal of cholesterol from cholesterol rich and cholesterol poor regions. Panel c shows the FCS autocorrelation data from the slow and faster regimes as highlighted in panel a, indicating an evolution of faster lipid

dynamics (see arrow) in '*F*' regime (panel, d) as compared to '*S*' regime (panel, c). In case of DMPC:Chl (1:1) bilayers, cholesterol has an expanding effect for the gel-like lipid DMPC, resulting in the loss of alkyl chain order (increased disorder) resulting in faster extraction from region *F* which we attribute to cholesterol-rich domains (*F*). Cholesterol is not easily removed from regions *S* where lipids are in a more ordered and cholesterol-poor environment. The above scenario is depicted schematically in Fig. S8e.

Figure S9a shows DOPC:Chl (3:1) confocal microscopy images as well as the line profile analyses (see ROI). The membrane disruption due to cholesterol extraction is more pronounced by uniformly reducing the intensity over the entire imaged region suggesting cholesterol at low concentration is loosely bound to the fluid-like lipid. The overall masking of intensity is completed before a period of ∼20 minutes as evidenced from the intensity drops down (blue line, right panel). In contrast to this, 50% cholesterol containing bilayers have revealed non-uniform reduction of intensity upon CD exposure (Fig. S9b) time. The reduction of intensity is more prominent in cholesterol poor regime ('*F*') than cholesterol rich ('*S*') regime (Fig. S9c) indicating the cholesterol rich domains are more stable due to the condensing effect of cholesterol.

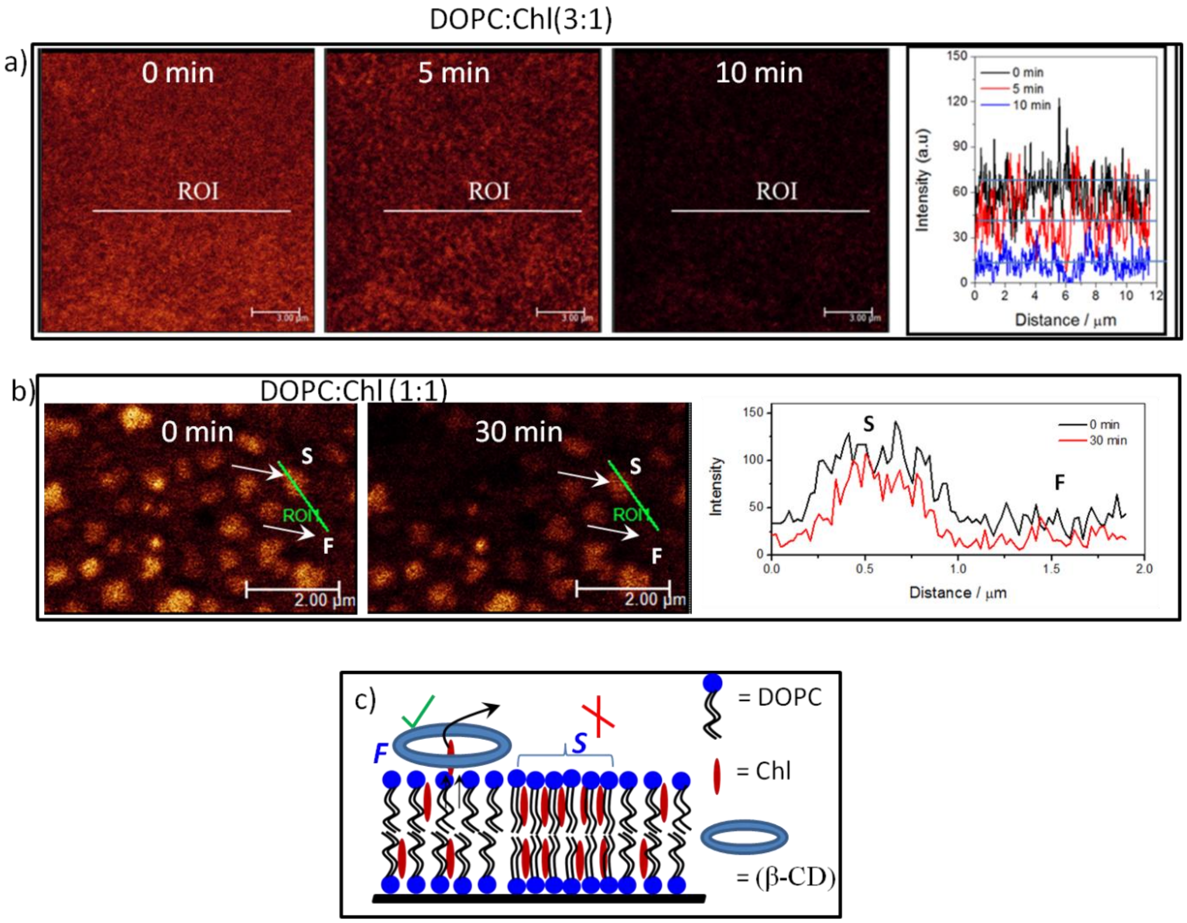

Figure S9. (a)Temporal evolution of confocal microscopy images of (a) DOPC:Chl (3:1) upon β-cyclodextrin treatment. The decrease in fluorescence intensity as marked in line (ROI) shown in the right panel. (b) Illustrate confocal microscopy image of DOPC:Chl(1:1) before (0 min) and after (30 min) β-cyclodextrin treatment. Right panel shows the decrease in fluorescence intensity across ROI; the reduction of intensity in '*F*' regime is more prominent than '*S*' regime. (c) Schematic representation of cholesterol removal from different spatial region (slow, *S* and fast, *F* regime).

In line with the confocal microscopy images of DOPC:Chl(1:1) bilayers, our FCS results also reveal the changes in diffusivity in these spatial regions. Differences in the temporal evolution of the transit times, τ_D between the different spatial regions, obtained from the in situ confocal FCS data (Figs. S10a and b) during CD experiments yields the information about the relative ease with which cholesterol can be extracted. The condensing effect of cholesterol in fluid-like DOPC lipids results in the formation of ordered cholesterol-rich domains (region *S*) from which

cholesterol extraction by CD is hindered. This results in a relatively invariant time evolution of τ_D upon exposure to CD over duration of 1 hr. In contrast, extraction of cholesterol from the cholesterol-poor region (*F*), is more rapid, reflecting the inherent fluidity in these domains. A rapid rise in diffusivity after 30 minutes in region *F* signals the removal of cholesterol (cf. Figure S10b). In the case of POPC, similar transit times from both regions *S* and *F* were observed over the duration of the CD experiment indicating that cholesterol was not sufficiently partitioned to create distinct domains (cf. Fig.S10c and d).

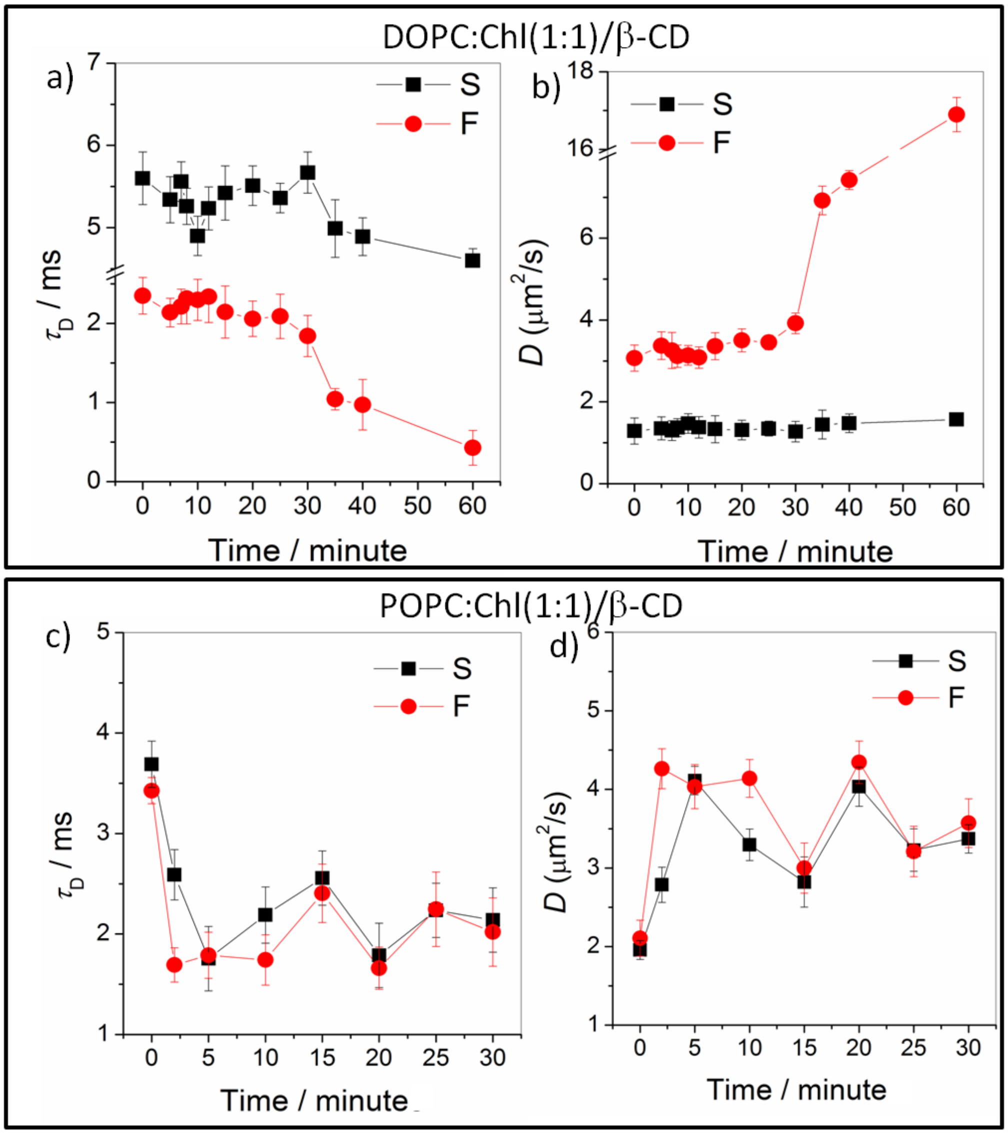

Figure S10. (a) and (c) shows the spatially resolved average τ_D versus time plot for DOPC and POPC lipidic system containing 50% cholesterol. (b) and (d) illustrate the respective time

evolution diffusivities. Note that the faster removal of cholesterol is more prominent from the *F* in the region DOPC:Chl bilayer compared to a slow removal from *S* region. In POPC:Chl bilayer the distinction between the *S* and *F* regions is minimal and the cholesterol depletion is similar from both the S and F regions.

Additionally, to understand the role of bulk lipid properties towards cholesterol removal, we have performed FCS measurement for DOPC, POPC and DMPC bilayer at 25% cholesterol content. In line with the confocal images as shown in Fig. S9a, the mobility in DOPC:Chl(3:1) bilayer greatly increases upon CD treatment as observed from the decrease in transit time (τ_D =2.2 to 1.43 ms) or increase in diffusivity (D =3.1 to 5 μm^2 s^{-1}) as shown in Fig. S11a and Fig. S11d and e (black circle). We further noticed that, upon prolonging the exposure time of CD to 30 minutes, the destabilization of membrane leads to removal of fluorescent phospholipid from the bilayer to the bulk as evidenced from the FCS trace (open gray in Fig. S11a), where the diffusivity is much faster and found to be 201 μm^2 s^{-1}. The change in diffusivity remains unaffected for POPC membrane (red circle) and the effect is moderate for DMPC (blue circle) bilayer as shown in Fig. S11e.

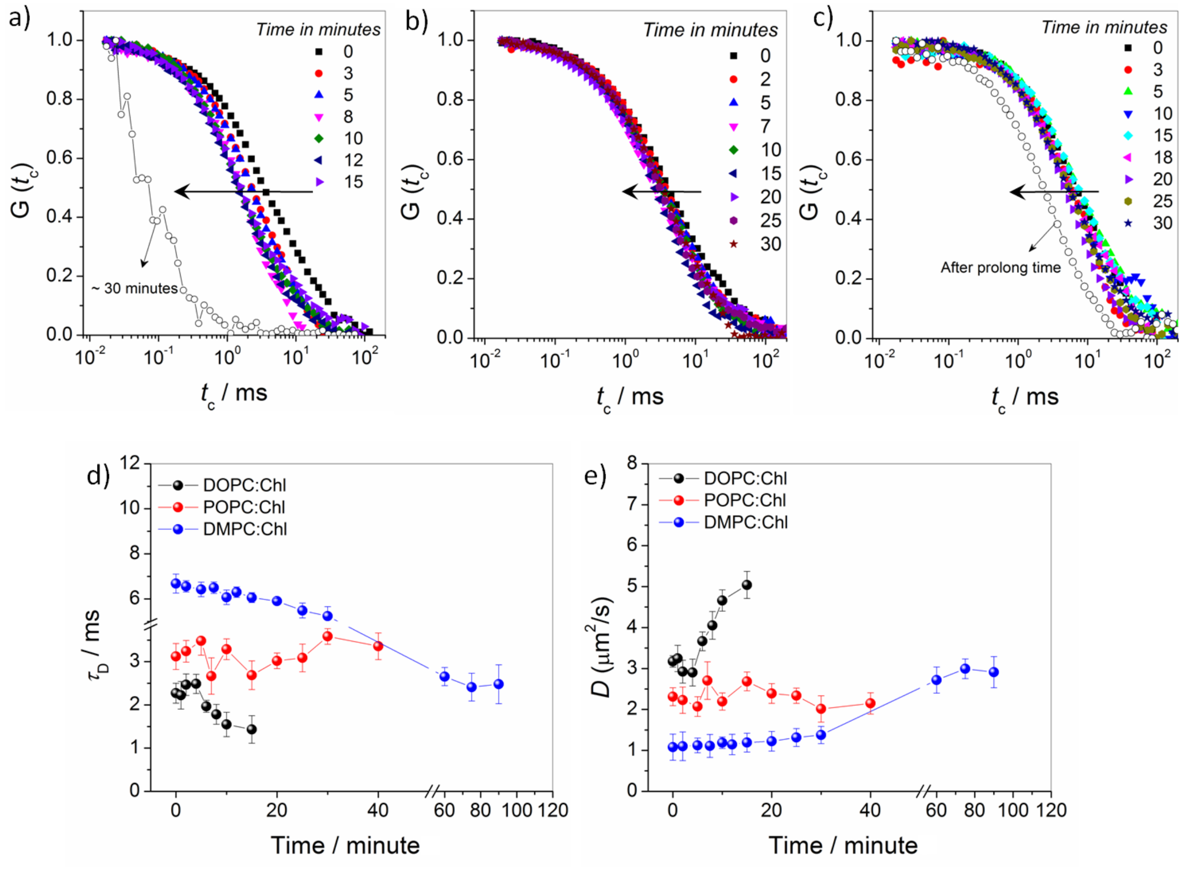

Figure S11. (a), (b) and (c) represents the autocorrelation data of diffusing fluorescent lipid acquired during the *in-situ* cholesterol depletion upon *β*-CD treatment in DOPC, POPC and DMPC bilayers containing 25% cholesterol each respectively. For clarity, few average curves are depicted. (c) and (d) shows the average τ_D and D versus time plot for DOPC and DMPC lipidic system, as determined from the fitting of the autocorrelation curves. Note that the faster removal of cholesterol is more prominent in DOPC:Chl bilayer.

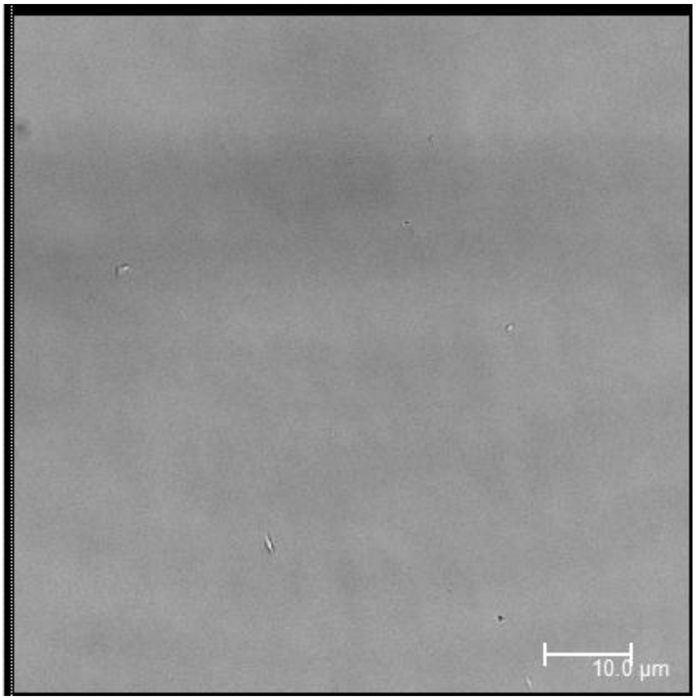

Figure S12. Differential interference contrast (DIC) microscopy image of DOPC:Chl(1:1) bilayer prepared by Langmuir-Blodgett technique show a homogeneous bilayer confirming the absence of cholesterol crystals.

Lipid dynamics on polymer cushioned bilayer:

Poly(acrylic acid) (PAA) cushioned were prepared (see methods in main text) and the thickness of the film was characterized by X-ray reflectivity (XRR) and atomic force microscopy technique (AFM). AFM (NT-MDT, Russia) measurements were performed in tapping mode with a cantilever of force constant ∼5 N m^{-1} and radius of curvature ∼8 nm. XRR measurement was carried out using Rigaku's SmartLab® diffractometer at Cu Kα (1.54 Å) radiation. The alignment of sample is completely automated with Rigaku's proprietary Guidance data acquisition software. X-ray reflectivity profiles depict variation of measured reflectivity R with the perpendicular momentum transfer, $q_z = \frac{4\pi}{\lambda}\sin\alpha$, where λ is the wavelength of the X-rays and α is the angle of incidence. The thickness (d) of the polymer was estimated by using the expression $d = 2\pi/\Delta q_z$ and found to be ~11.6 nm (see Fig. S13c) which is consistent with the height profile analysis from AFM measurement (see Fig. S13b).

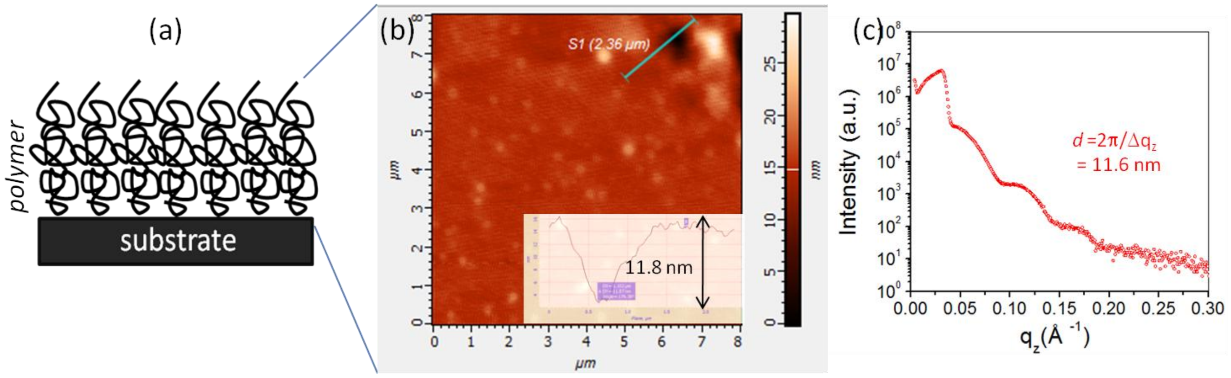

Figure S13. Schematic representation of poly (acrylic acid) (PAA) cushion. (b) Topographic AFM image of PAA on glass substrate prepared by spin coating method. Inset shows the line profile showing a thickness of 11.8 nm. (c) X-ray reflectivity data (thickness, d = 11.6 nm) of PAA cushion on glass substrate.

In the presence of PAA cushion, at low concentration of cholesterol content (25%), the DOPC-Chl bilayer is homogeneous (Fig. S14a) and FCS diffusion law reveals Brownian diffusion ($t_0 = 0$) with a diffusivity value which is slightly less than the uncushioned bilayer (cf. Fig. S14b).

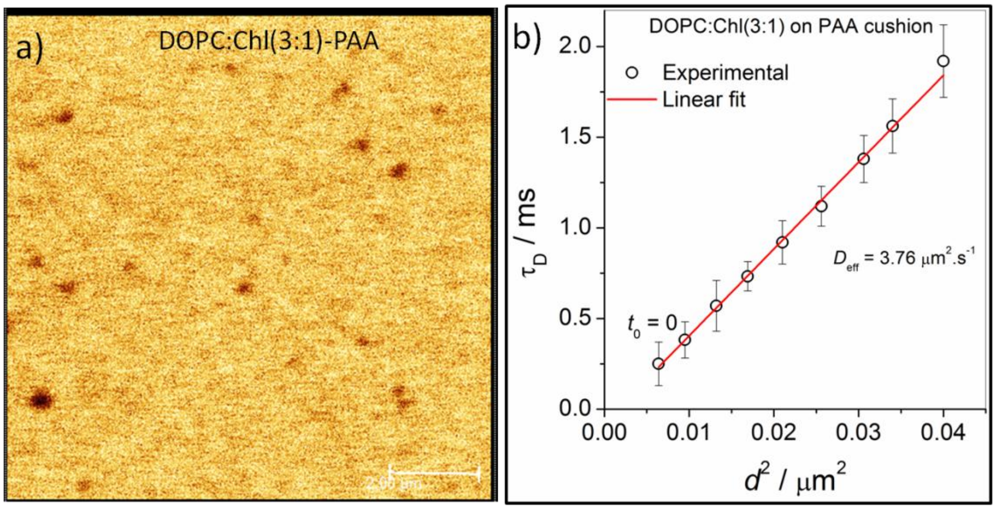

Figure S14. (a) Confocal microscopy images of DOPC:Chl(3:1) bilayer on PAA cushion illustrating largely uniform fluorescence intensity. The bilayer was stained with Atto488 PE (5×10^{-4} mol%). The scale bar is 2 µm. (b) Dependence of transit time (τ_D) versus d^2 plot acquired from the PAA cushion DOPC:Chl(3:1) bilayer showing the free Brownian diffusion ($t_0 = 0$).

At 50% cholesterol content, DOPC and DMPC bilayers, reveal a heterogeneous distribution of fluorescence intensity in the confocal images and a bimodal diffusivity (Fig. S15, SI). In cushioned platform, the differences in diffusivity values between the two domains are slightly different than that of the supported bilayers prepared on the glass substrates in the absence of cushion. From the FCS diffusion law data in STED mode, we also observed a crossover in diffusion behavior in these two morphologically distinct domains like the trends observed for bilayers on glass substrate (cf. Fig. S16, SI). The negative intercept values were found to be -2.5 and -1.9 for DOPC:Chl(1:1) and DMPC:Chl(1:1) bilayers respectively in slow phase. The estimated domain size (ω) in the respective bilayers were found to be 91±2 and 94±3 nm.

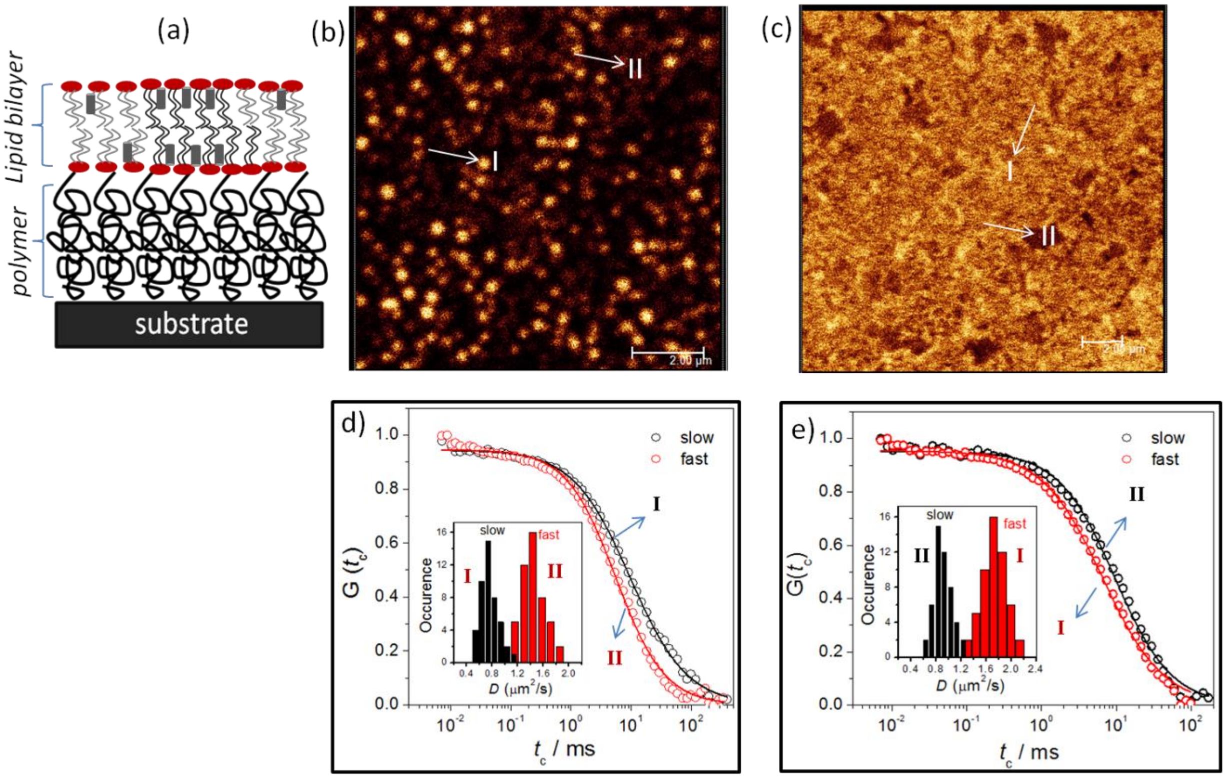

Figure S15. (a) Schematic representation of polymer cushioned bilayer. (b) and (c) illustrate the confocal microscopy images of DOPC:Chl(1:1) and DMPC:Chl(1:1) bilayer on PAA cushion respectively. Panel (d) and (e) represent the time scales of lipid diffusion in confocal mode at spatial points I and II as marked in Figs. b and c. The autocorrelation function (G(t_c)) is plotted as a function of lag time (t_c) and the autocorrelation curve is fitted with non-linear least square fit

(solid lines) method using equation 1 (see main manuscript). Inset in the panel d and e represent the frequency plot of the diffusivity values from the respective spatial points. The diffusivity data are collected from ~30-50 measurements from three independent samples.

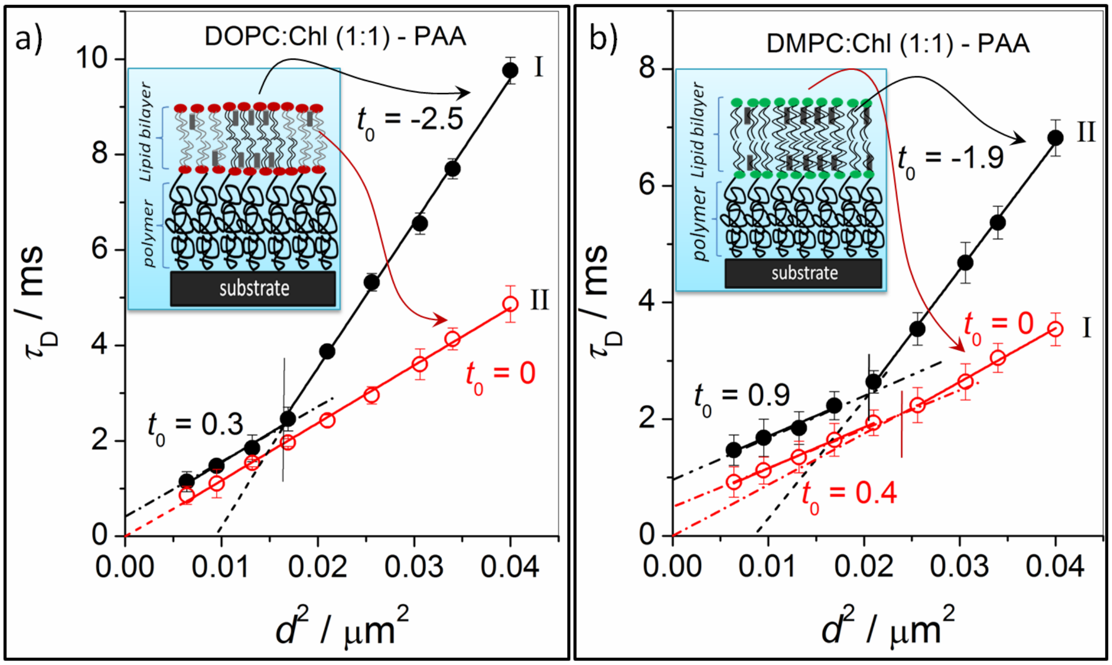

Figure S16. (a) and (b) represents the FCS diffusion law plots for DOPC:Chl(1:1) and DMPC:Chl(1:1) bilayers respectively on PAA cushion. The slow and fast regimes are represented by closed and open symbol. Solid lines are the linear fit and dotted lines are the extrapolated lines showing the intercept values (t_0) in each regime. The vertical solid line, in respective panels, indicates the crossover length scale, ξ, between two dynamical regimes characterized by free or hindered lipid diffusion. Inset shows the schematic representation of PAA cushioned bilayer and the curved arrow represents the spatially resolved dynamics from the cholesterol poor and rich domains.
